# Genotoxic, Cytotoxic, and Physiological Effects of Nano- and Microplastics in Invertebrate Model Organisms: Mechanistic Integration, Adverse Outcome Pathways, and Multi-Omics Perspectives

**DOI:** 10.3390/toxics14080666

**Published:** 2026-07-28

**Authors:** Ahmet Ali Berber, Cansu Akbulut

**Affiliations:** 1 Vocational School of Health Services, Çanakkale Onsekiz Mart University, 17100 Canakkale, Türkiye; aberber@comu.edu.tr; 2 Department of Biology, Faculty of Science, Sakarya University, 54050 Sakarya, Türkiye

**Keywords:** nanoplastics, microplastics, invertebrate, genotoxicity, oxidative stress, adverse outcome pathway, multi-omics, epigenetic toxicity

## Abstract

Nano- (NPs, <1 µm) and microplastics (MPs, 1 µm–5 mm) are ubiquitous contaminants whose toxicity to invertebrates carries implications at the individual scale and (although not yet quantitatively validated) at the population scale. This semi-systematic narrative review organizes available evidence within an adverse outcome pathway (AOP) framework, linking primary molecular initiating events (MIEs) to adverse outcomes while flagging evidence strength at each step. It involves a structured synthesis that applies selected PRISMA 2020 transparency principles, namely disclosed databases, a priori eligibility criteria, and explicit harvest and de-duplication counts, but does not attempt the exhaustive paired screening, formal risk-of-bias scoring, or quantitative meta-analysis of a full systematic review; this design was chosen because the marked heterogeneity of particle physicochemistry, exposure regimes, and endpoint metrics across the available literature makes pooled statistical synthesis premature. Evidence is appraised across *Daphnia*, *Artemia*, *Chironomus*, *Caenorhabditis elegans*, *Eisenia*, marine mollusks, and crustaceans. Polymer chemistry, size, surface charge, weathering, biofilm formation, additives, and adsorbed co-contaminants shape uptake and downstream toxicity. Reactive oxygen species, mitochondrial dysfunction, lysosomal destabilization, and ER stress recur as coupled key events downstream of four MIEs (direct membrane interaction, protein corona formation, surface-catalyzed redox chemistry, and Trojan horse delivery). Multi-omics datasets converge on dysregulated stress, repair, apoptotic, immune, and inflammatory programs; epigenetic marks are increasingly considered as substrates for persistent and potentially heritable toxicity, although stable transgenerational transmission remains poorly demonstrated. The review delivers (i) an AOP map with evidence-strength annotations (strong, moderate, emerging), (ii) a structured cross-study synthesis comparing NP and MP effect profiles, and (iii) a critical layer that reinterprets biphasic and apparently contradictory data as mechanistically informative once particle physicochemistry and tissue context are resolved. Progress will depend on standardized characterization, environmentally realistic mixtures, and AOP-anchored designs that distinguish experimentally demonstrated mechanisms from inferred mechanisms and theoretical extrapolations.

## 1. Introduction

Annual global plastic production now exceeds 400 million tonnes, and a sizable fraction enters terrestrial and aquatic systems through mismanaged waste streams [1,2]. Once released, plastic items break down via UV photodegradation, mechanical abrasion, thermo-oxidative reactions, and microbial action. The result is a continuum of secondary microplastics and nanoplastics that complements the primary fragments produced by industry [3,4,5]. These particles have been recovered from deep-sea sediments, polar ice, atmospheric deposition, freshwater systems, agricultural soils, and animal tissues across virtually every taxon investigated [6,7,8,9]. Detection in human blood, placenta, lung, and brain underscores their reach across the biosphere [10,11,12].

Invertebrates occupy a uniquely informative position within this contamination landscape. They make up the bulk of described animal diversity, drive nutrient cycling, support pollination and decomposition, and form the prey base for higher trophic levels. As primary consumers, detritivores, filter feeders, and prey items, invertebrates are also the principal entry route by which NPs and MPs propagate through food webs. Their physiological responses are widely used as early indicators of pollutant stress, although the extent to which individual-level biomarker shifts translate into community- and ecosystem-level disturbance is generally inferred rather than demonstrated and remains to be validated for plastic particle exposures specifically [13,14]. Short generation times, transparent or simple body plans, well-annotated genomes, and amenability to high-throughput experimentation have made species such as *Daphnia magna*, *Caenorhabditis elegans*, *Mytilus galloprovincialis*, and *Eisenia fetida* indispensable for mechanistic ecotoxicology and for animal-focused environmental risk assessment [15,16,17,18]. Empirical work shows that NP/MP exposure (even at environmentally realistic concentrations) can disturb redox balance, damage the genome, dysregulate gene networks, impair reproduction, and produce phenotypes that, in some experimental systems, persist into offspring generations [19,20,21,22,23,24]. Vertebrate ecotoxicological consequences are treated separately in the literature; in particular, the toxicokinetic and toxicodynamic responses of amphibians and reptiles to MP exposure, including oxidative stress cascades, endocrine-axis disruption, microbiome dysbiosis, and immune compromise, have recently been synthesized in a companion review [25], and the present synthesis is intended to complement rather than overlap with that vertebrate-focused treatment.

Despite rapidly growing literature, the field remains methodologically heterogeneous and conceptually fragmented. Studies differ in particle characterization, exposure design, and endpoint selection, which makes synthesis difficult [26,27]. Individual endpoints, genotoxicity, oxidative stress, and apoptosis are well documented, but integrative analyses linking molecular initiating events to organismal and population-level outcomes remain rare [28,29]. High-throughput omics platforms and the formalization of the adverse outcome pathway (AOP) framework now make such integration tractable [30,31].

**Figure 1:**
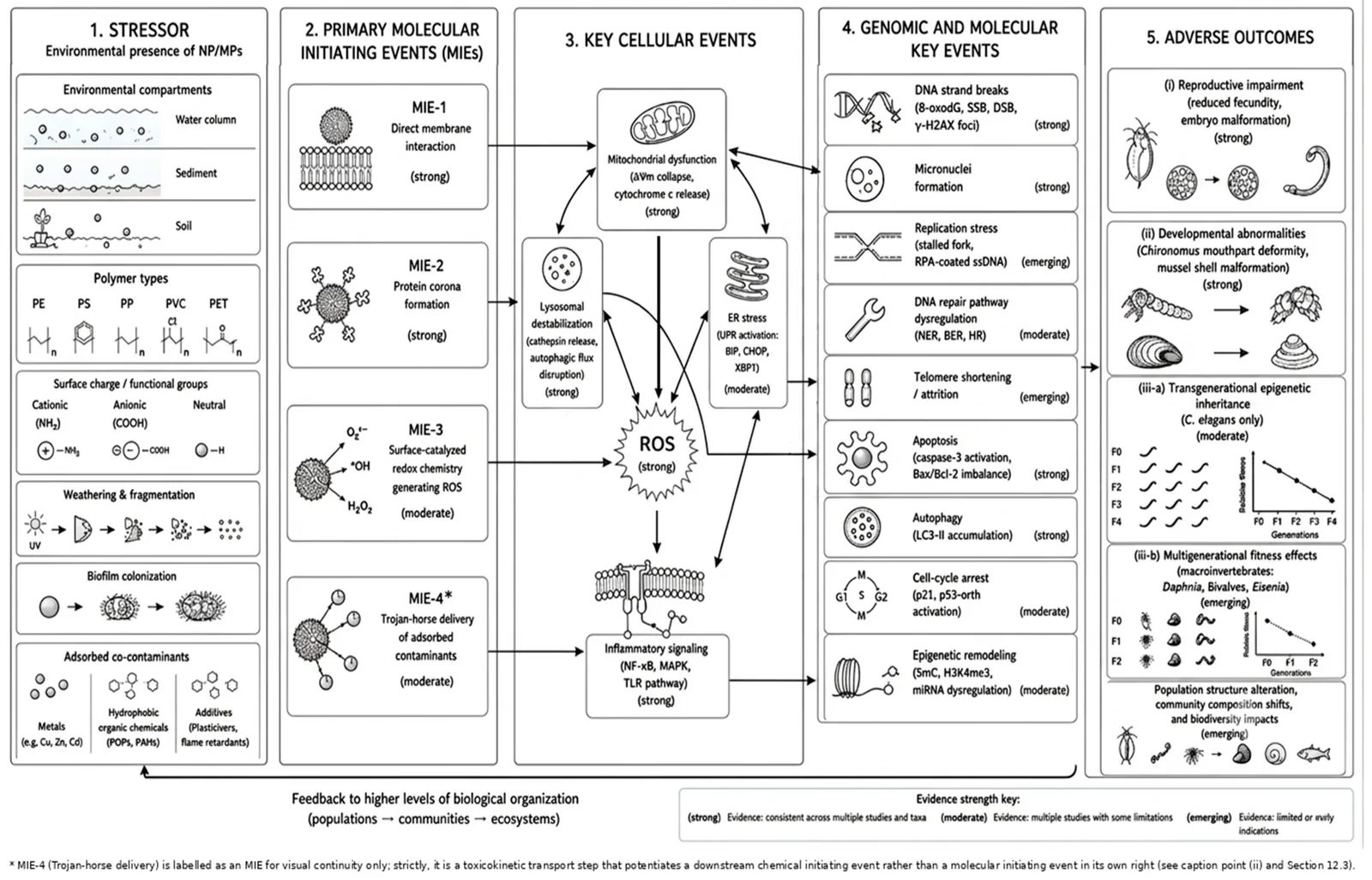
Adverse outcome pathway (AOP) framework for NP/MP toxicity in invertebrates. Schematic mapping the proposed causal chain from primary molecular initiating events (MIEs), direct membrane interaction, protein corona formation, surface-catalyzed redox chemistry, and Trojan horse co-contaminant delivery, through key cellular and tissue-level events (ROS generation, mitochondrial and lysosomal dysfunction, ER stress, DNA damage and DNA damage response engagement, inflammatory signaling, and epigenetic remodeling) to individual-level adverse outcomes that are robustly documented (impaired reproduction, developmental abnormalities, behavioral deficits) and to taxonomically differentiated multi- and transgenerational fitness outcomes—(iii-a) transgenerational epigenetic inheritance restricted to *Caenorhabditis elegans*, for which evidence is currently moderate, and (iii-b) multigenerational (F0–F2) fitness effects in macroinvertebrates (*Daphnia*, marine bivalves, *Eisenia* spp.), for which evidence is emerging together with inferred but currently unvalidated consequences for community structure. Pathway components are color-coded by AOP tier (MIE, key event, adverse outcome) and shaded by qualitative evidence strength (strong, moderate, emerging) as defined in Section 12. The revised figure incorporates three interpretive clarifications: (i) reactive oxygen species are shown as a frequently engaged but non-obligatory node, with parallel ROS-independent routes (mechanical injury, membrane wrapping, lysosomal/ER stress, and additive- or co-contaminant-mediated effects) able to reach downstream key events without passing through ROS; (ii) Trojan horse delivery is depicted as a toxicokinetic transport step that potentiates a downstream chemical initiating event (for example, receptor binding or enzyme inhibition by the delivered contaminant) rather than as a molecular initiating event in its own right (the MIE-4 label in the graphic accordingly carries an asterisk, MIE-4*, that refers to a direct footnote at the bottom of the figure formalizing this compromise); and (iii) the previous single “multigenerational and transgenerational fitness loss” node has been split into the two taxonomically distinct outcomes (iii-a) and (iii-b) described above, so that causally interrogated transgenerational evidence is not conflated with emerging multigenerational evidence (see Section 12.6).

Positioning and novelty of this review. Several earlier reviews surveyed NP/MP toxicity in aquatic biota [28,32,33,34]. Most adopt an endpoint-by-endpoint structure, with elevated comet tail moments, induced antioxidant enzymes, and altered *hsp70* transcripts without an explicit causal chain. The present review departs from that tradition in three ways. First, evidence is organized around an AOP scaffold with explicit evidence-strength annotation (strong, moderate, emerging) for MIEs, key cellular events, and adverse outcomes; this lets readers follow causal logic rather than parallel taxonomies. Second, a analysis layer (Section 14) treats biphasic antioxidant responses, opposing methylation directions, and contradictory micronucleus data as mechanistically interpretable signals once particle physicochemistry, dispersion stability, and tissue specificity are resolved. Together, these features convert a literature catalog into a mechanistic synthesis with implications some demonstrated, and others hypothesis-generating for invertebrate biology, biodiversity-relevant monitoring, and ecological risk assessment (Figure 1).

On that basis, we synthesize current evidence on the genetic, cytotoxic, and molecular consequences of NP/MP exposure in invertebrate models. We dissect the pathways that link particle physicochemistry to biological outcomes, evaluate emerging biomarkers and omics-based readouts, and critically appraise methodological limits and research priorities. The aim is to provide a mechanistic foundation for the next phase of plastic ecotoxicology, while explicitly delineating where current evidence supports robust inference at the population or ecosystem scale and where such extrapolation remains theoretical.

## 2. Literature Search Strategy and Selection Criteria

This work is presented as a semi-systematic narrative review: a literature synthesis that adopts selected PRISMA 2020 [35] transparency principles, database disclosure, structured search strings, a priori eligibility criteria, and explicit reporting of harvest and de-duplication counts, without claiming the methodological completeness of a formal systematic review or meta-analysis. The objective was mechanistic integration of the genotoxic, cytotoxic, and physiological effects of NPs and MPs in invertebrate models, with priority given to evidence amenable to AOP construction and multi-omics interpretation. Quantitative effect-size synthesis was not attempted: the heterogeneity of particle physicochemistry, exposure regimes, endpoint metrics, and reporting conventions across the available literature makes pooled statistical synthesis prone to spurious precision rather than informative. Risk-of-bias was appraised qualitatively rather than scored numerically, in line with the narrative review design. A semi-systematic rather than fully systematic design was chosen deliberately: at the present stage of NP/MP ecotoxicology, particle characterization and endpoint metrics remain too heterogeneous and inconsistently reported for paired independent screening, formal risk-of-bias scoring, and meta-analytic pooling to add reliable inferential value, so a transparent, mechanistically prioritized synthesis is better matched to the maturity of the evidence base.

### 2.1. Databases and Search Date

Three primary bibliographic databases were searched: Web of Science Core Collection (Clarivate, Philadelphia, PA, USA; SCI-EXPANDED + SSCI indices), Scopus (Elsevier, Amsterdam, The Netherlands), and PubMed/MEDLINE (NCBI, Bethesda, MD, USA). Google Scholar (Google LLC, Mountain View, CA, USA) was used as a secondary source restricted to the retrieval of gray literature and citing references not surfaced by the indexed databases, and was not used as a primary evidence base because of its limited reproducibility and lack of stable export functions. The search was executed on 11 May 2026 as a single comprehensive harvest, covering publications indexed up to and including 30 April 2026. The primary search window was set as 1 January 2010–30 April 2026; no records satisfying the full Boolean string appeared in Web of Science before 2013 or in Scopus before 2015, reflecting the recent emergence of “nanoplastic” and “microplastic” as indexed terms in those databases. PubMed records were available from 2010 onward. Earlier seminal studies (e.g., on AOP conceptualization [30] and nanoparticle uptake principles [36,37]) were retained outside this Boolean harvest, where they provided indispensable mechanistic anchoring; these exceptions are flagged at the relevant in-text citations.

### 2.2. Search Strings and Database-Specific Syntax Harmonization

Boolean search strings combined three concept blocks. Block A (particles): “nanoplastic*” OR “microplastic*” OR “polystyrene nanoparticle*” OR “PS-NP*” OR “PE microplastic*” OR “PVC microplastic*”. Block B (biological endpoint): “genotoxic*” OR “DNA damage” OR “comet assay” OR “micronucleus” OR “oxidative stress” OR “apoptosis” OR “autophagy” OR “transcriptom*” OR “proteom*” OR “metabolom*” OR “epigenet*” OR “miRNA” OR “transgenerational” OR “mitochondrial dysfunction” OR “ER stress”. Block C (organism): “invertebrate*” OR “*Daphnia*” OR “*Artemia*” OR “*Chironomus*” OR “*Caenorhabditis elegans*” OR “*Eisenia*” OR “*Mytilus*” OR “earthworm*” OR “bivalve*” OR “crustacean*”. Records were retained at the Boolean intersection (A AND B AND C). The base string was harmonized to the indexing syntax of each database before execution: in Web of Science, the string was applied to the Topic (TS) field, which queries title, abstract, and author keywords; in Scopus, to the TITLE-ABS-KEY field; and in PubMed/MEDLINE, with simplified quotation of multi-word phrases and reliance on PubMed automatic term mapping to expand controlled vocabulary where applicable. Asterisk truncation was used in Web of Science and Scopus; PubMed automatic term mapping handled lexical variants without explicit wildcards in most fields. Citation snowballing (backward) was applied during the construction of subsection-specific syntheses to recover foundational and mechanistically critical references that predated the 2010 cutoff or appeared in journals not fully indexed by the three primary databases.

### 2.3. Eligibility Criteria

Inclusion required that a record (i) reported original empirical findings or peer-reviewed synthesis on NP/MP exposure to invertebrate models; (ii) described at least one molecular, cellular, genotoxic, omics, reproductive, or behavioral endpoint; (iii) provided sufficient methodological detail to evaluate particle characterization and exposure conditions; and (iv) was published in a peer-reviewed venue in English. Foundational reviews and methodological papers on AOPs, comet assay validation, and nano–bio interactions were also retained for mechanistic anchoring, even when outside the principal time window. Exclusion criteria removed records that (i) addressed only vertebrate or microbial systems; (ii) reported physicochemical characterization without biological endpoints; (iii) lacked controls or quantitative outcome reporting; (iv) exclusively addressed the engineering, recycling, or remediation of plastics; or (v) appeared only as conference abstracts not subsequently published in full, non-peer-reviewed reports, or retracted articles. Eligibility criteria were specified before screening commenced and were not modified during the screening process.

### 2.4. Duplicate Removal and Screening Workflow

Records exported from the three primary databases (in RIS format from Web of Science and Scopus; in NBIB format from PubMed) were imported into Zotero (Corporation for Digital Scholarship, Vienna, VA, USA), where duplicates were removed in two passes: an automated DOI and title matching pass, followed by a manual review of records flagged as near duplicates by partial title or partial author matches (typically arising from differences in journal name formatting between databases or from preprint–publication pairs). The harvest returned 956 records from Web of Science (publication years 2013–2026), 1059 from Scopus (2015–2026), and 627 from PubMed/MEDLINE (2010–2026), for a combined raw total of 2642 records; the asymmetry in start years reflects database-specific indexing of “nanoplastic” and “microplastic” terms rather than a filter setting. Two-pass de-duplication eliminated 1346 redundant records, leaving 1296 unique records (a duplication rate of approximately 51%, consistent with the substantial inter-database overlap expected for this topic). From this de-duplicated corpus, the present review draws on a focused, mechanistically prioritized subset of 188 studies reflecting the authors’ expert judgment of relevance to the AOP-anchored synthesis presented here, supplemented by 10 additional 2022–2025 records identified through targeted topic-specific searches during manuscript revision to address citation density gaps in specific subsections (replication stress, telomere endpoints, multi-omics integration, transgenerational effects in bivalves, AOP frameworks, and quality criteria guidance). We emphasize that the 188 records reflect a curated subset selected by joint consensus review of the two authors (see Section 2.5) rather than the output of an exhaustive title/abstract and full text screening of all 1296 unique records; this is a defining feature of the semi-systematic narrative design and is explicitly addressed under limitations (Section 2.7).

### 2.5. Study Selection Procedure

Because this is a semi-systematic narrative review rather than a formal systematic review, study inclusion did not follow a paired screener PRISMA workflow with independent dual screening. Instead, the de-duplicated corpus (1296 records) was reviewed jointly by the two authors in shared working sessions, with each candidate record assessed against the a priori eligibility criteria (Section 2.3) and retained or excluded by consensus at the time of review. This joint review approach was chosen deliberately for a narrative synthesis of a heterogeneous, mechanistically oriented literature, in which judgment about the relevance of a study to AOP construction and multi-omics integration benefits from real-time discussion between reviewers more than from post hoc reconciliation of independent decisions. Records on which the two authors did not immediately agree at first review were set aside and revisited after rereading the full text, with a final decision reached by consensus against the eligibility criteria. We did not compute formal inter-rater agreement statistics (e.g., Cohen’s κ), because such statistics are defined for paired independent screening and are not meaningful for a consensus review workflow in which decisions are made jointly rather than in parallel; we treat this explicitly as a feature of the narrative review design (Section 2.7) rather than as a metric the workflow could have supported.

### 2.6. Data Extraction and Qualitative Quality Appraisal

From each included primary study, the two authors jointly extracted the following items into a structured table: (a) species and life stage; (b) polymer type, size, surface modification, and weathering state; (c) exposure medium, concentration, and duration; (d) endpoints assessed (genotoxic, cytotoxic, oxidative, molecular, omics, reproductive, behavioral, transgenerational); (e) principal effect direction and magnitude as reported by the authors of the primary study; (f) reported particle characterization detail (hydrodynamic size, zeta potential, polydispersity index, where given); and (g) reference identifier. Extracted data populated the structured comparative synthesis presented in Table 1. A simplified qualitative quality framework adapted from de Ruijter et al. [27] and Jemec Kokalj et al. [38] was applied to each primary study, considering four domains: particle characterization completeness, dispersion stability assessment, exposure realism, and endpoint reporting transparency. These domains were used to weight interpretive emphasis (in particular, in the critical analysis layer of Section 14) rather than to assign numerical risk-of-bias scores. Studies with weak characterization or poorly documented dispersion were retained but flagged in context. This approach is appropriate to a mechanistic narrative review of a heterogeneous literature; it is not equivalent to the formal risk-of-bias scoring (e.g., ROBINS-I, GRADE) expected of a Cochrane-style systematic review, and we make no such equivalence claim.

### 2.7. Limitations of the Semi-Systematic Narrative Methodology

We explicitly note five limitations of the present methodology that bear on interpretation. First, the restriction to English language peer-reviewed publications excludes a non-trivial body of relevant work published in Chinese, Spanish, and other languages, and may underweight regionally focused exposure studies. Second, the prioritization of mechanistically informative endpoints (genotoxicity, oxidative stress, omics, transgenerational effects) means that purely behavioral or purely ingestion quantification studies are under-represented in our included set relative to their share of the de-duplicated corpus. Third, study selection from the 1296 unique records to the 188 retained for synthesis was conducted by joint consensus review between the two authors rather than by independent paired screening. The corresponding absence of an inter-rater agreement metric (e.g., Cohen’s κ) is an intrinsic and deliberate consequence of this consensus review design, in which decisions are made jointly rather than in parallel, and is reported transparently here rather than addressed with a metric the workflow does not support. Fourth, no formal risk-of-bias scoring (e.g., ROBINS-I, GRADE) was applied at the included study level; quality appraisal is qualitative, organized around the four domains described in Section 2.6. Fifth, the decision not to perform quantitative meta-analysis means that the strength of cross-study effects is presented qualitatively (Table 1) rather than via pooled effect sizes. We regard these limitations as appropriate trade-offs for a mechanistic synthesis of a heterogeneous, methodologically inconsistent literature; full systematic review methodology with paired screening, formal risk-of-bias scoring, and meta-analysis is, in our judgment, premature for this field at present and would require an upstream effort to standardize particle characterization and endpoint metrics before it could meaningfully proceed.

### 2.8. Synthesis Approach

Findings were organized thematically rather than chronologically, with explicit attention to convergent evidence across taxa, to mechanistically informative outliers, and to apparently conflicting results that resolve when particle physicochemistry, dispersion behavior, or exposure regime are properly considered (see Section 14). Particle physicochemistry, exposure realism, and study quality were used to weight interpretive emphasis throughout. The structured tabular synthesis (Table 1) supports transparent cross-study comparison (including explicit annotation of exposure realism and qualitative evidence strength per study) and is intended to guard against unsupported generalization across taxa, polymer types, and exposure scales.

## 3. Sources, Characteristics, and Environmental Fate of Nano and Microplastics

The toxicological behavior of plastic particles cannot be separated from their physicochemical identity, which is itself shaped by environmental processing. Polyethylene (PE), polypropylene (PP), polystyrene (PS), polyvinyl chloride (PVC), and polyethylene terephthalate (PET) account for most environmental plastic debris. Each polymer carries distinct densities, glass transition temperatures, monomer compositions, and weathering kinetics [65,66]. Buoyant polymers such as PE and PP dominate surface waters, while denser polymers such as PVC and PET concentrate in sediments where benthic invertebrates encounter them at elevated levels [67]. Polymer-specific monomer toxicity (e.g., styrene from PS, vinyl chloride from PVC) overlays an intrinsic chemical hazard on the physical hazard of the particle. This distinction is often blurred in particle-centric exposure designs [68,69].

Particle size is the most decisive driver of biological reactivity. Particles below ~100 nm have very high surface area to volume ratios. They are highly reactive, form protein coronas readily, and enter cells through endocytic and direct membrane translocation pathways [70,71]. Microplastics are typically internalized through ingestion. Their effects arise from mechanical abrasion of digestive epithelia, gut microbiome disruption, and slow leaching of additives [72,73]. Surface charge further modulates behavior. Cationic, amine-functionalized PS particles consistently produce greater membrane disruption, mitochondrial depolarization, and cytotoxicity than anionic carboxylate or neutral counterparts. The pattern is driven by electrostatic interaction with negatively charged biological membranes and lysosomal compartments [74,75].

Environmental aging fundamentally alters NP/MP toxicity. Photooxidation, thermooxidation, and mechanical fragmentation introduce oxygen-containing functional groups (carbonyl, hydroxyl, carboxyl), increase surface roughness, and accelerate the leaching of monomers, oligomers, and additives [76,77]. Weathered particles therefore display elevated ROS-generating capacity, stronger sorption affinity for hydrophobic organic contaminants, and enhanced bioavailability relative to pristine counterparts [78,79,80]. Biofilm colonization (the so-called “plastisphere”) adds another layer of complexity. Biofilms alter buoyancy, surface chemistry, and palatability to grazers, and can act as vectors for opportunistic pathogens or antibiotic resistance genes that propagate through invertebrate consumers [81,82].

Plastic matrices act as reservoirs for weakly bound chemical additives (phthalate plasticizers, brominated flame retardants, bisphenols, organotin stabilizers, antioxidants) that leach into surrounding media or biota with documented endocrine-disrupting and genotoxic potential [68,69,83]. The high specific surface area of NPs and MPs also enables sorption of co-occurring contaminants (metals, pesticides, pharmaceuticals, antibiotics), generating “Trojan horse” complexes whose combined toxicity often exceeds that predicted from additive single compound effects [84,85,86]. Mechanistic ecotoxicology must therefore treat NPs and MPs not as discrete chemicals but as dynamic, multi-component vectors whose biological effects emerge from the integrated action of polymer chemistry, morphology, weathering state, surface ecology, and contaminant load.

## 4. Uptake, Internalization, and Bioaccumulation in Invertebrates

Uptake routes are size and species-specific. In filter-feeding zooplankton such as *Daphnia magna* and bivalves such as *Mytilus* spp., particles enter with food and water and accumulate in the digestive tract, hepatopancreas, and gills [36,87]. *Daphnia* show pronounced size selectivity, ingesting particles in the 0.1–10 µm range most efficiently. Smaller particles preferentially translocate across the gut epithelium into the haemolymph [88]. In *C. elegans*, a soil and microbial feeding nematode, particles are ingested via pharyngeal pumping and accumulate in the intestinal lumen. Smaller PS nanoparticles can cross the gut barrier and reach reproductive tissues [44,45]. Earthworms such as *Eisenia fetida* and *Eisenia andrei* encounter MPs through dermal contact and ingestion of contaminated soil and litter, with particles accumulating in coelomic fluid, intestinal epithelium, and chloragogenous tissue [54,89].

At the cellular level, NPs cross plasma membranes via clathrin- and caveolae-mediated endocytosis, macropinocytosis, and (for the smallest particles) direct diffusion through lipid bilayers [37,90]. Internalized particles are then trafficked predominantly to lysosomes. Acidic pH and proteolytic activity can destabilize their structure, release adsorbed contaminants, and trigger lysosomal membrane permeabilization [91,92]. The protein corona that forms upon contact with biological fluids modulates cellular recognition, uptake kinetics, and intracellular fate [93,94]. Bioaccumulation is often transient owing to depuration. Repeated or chronic exposure, however, leads to sustained tissue burdens, particularly in long-lived life stages and species with limited excretory capacity [95,96].

**Figure 2:**
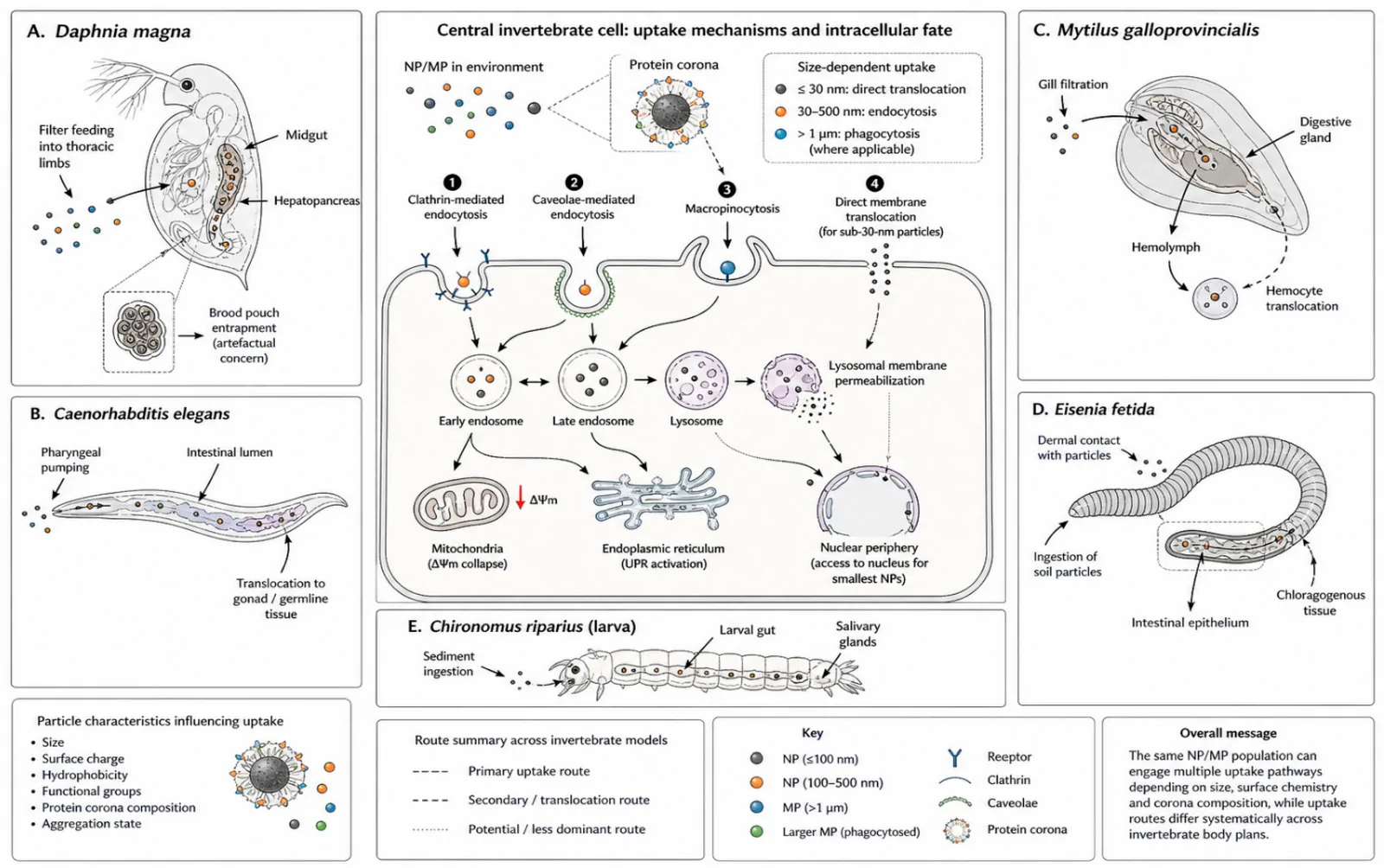
Uptake and internalization pathways of NPs and MPs across representative invertebrate models. Particle entry routes are shown for filter-feeding zooplankton (*Daphnia* spp.), bivalves (*Mytilus* spp., *Crassostrea gigas*), nematodes (*C. elegans*), oligochaetes (*Eisenia* spp.), and crustaceans (*Artemia* spp.), highlighting size- and species-specific routes (gill filtration, gut ingestion, dermal contact, pharyngeal pumping). At the cellular level, the schematic outlines the principal internalization mechanisms relevant to NPs (clathrin- and caveolin-mediated endocytosis, macropinocytosis, and direct translocation of sub-100-nm particles) and to MPs (phagocytic uptake by hemocytes and coelomocytes, and trans-epithelial transport). Tissue distribution is indicated for the digestive tract, hepatopancreas, gonads, hemolymph, and reproductive tissues. Particle characteristics (size, charge, weathering state, biofilm corona) modulate both uptake efficiency and the dominant downstream pathway, as discussed in Section 4. This cellular schematic is a deliberate conceptual generalization and does not represent a universal invertebrate cell. Filter-feeding bivalves, pharyngeal-pumping nematodes, dermally and orally exposed earthworms, and limb-filtering crustaceans differ substantially in immune architecture, metabolic organization, and genome biology, so the entry routes and downstream molecular outcomes shown should be read as taxon-dependent rather than interchangeable.

Taxon comparison (bioaccumulation and human-health relevance). The magnitude and toxicological significance of NP/MP bioaccumulation differ systematically among invertebrate groups. Sessile, filter-feeding bivalves process large volumes of water across their gills and are therefore particularly susceptible to particle accumulation; because mussels, oysters, and clams are frequently consumed whole, and sometimes raw, their tissue burdens have direct relevance for human dietary exposure and seafood safety. Decapod crustaceans, by contrast, integrate exposure across compartments. Freshwater crayfish in particular absorb contaminants from water, sediment, and diet, and their hepatopancreas, gills, muscle, and exoskeleton accumulate metals, organic pollutants, and microplastics in tissue-specific patterns, making them informative integrative bioindicators of aquatic pollution and of associated seafood safety hazards [97]. Nematodes such as *C. elegans* and earthworms such as *Eisenia* spp. accumulate particles primarily through pharyngeal ingestion and through combined dermal contact and ingestion of contaminated soil, respectively, so their body burdens reflect sediment- and soil-phase bioavailability rather than aqueous filtration [44,45,54,89]. Body burden, target tissue, and downstream risk are therefore not directly comparable across taxa, and the choice of model should be matched to the exposure compartment of interest (Figure 2).

### 4.1. Comparative Mechanisms: Nanoplastics Versus Microplastics

The contrast between nano and microscale plastic particles is not merely a question of dimension. It reflects fundamentally different routes of biological engagement, intracellular fate, and dose–response architecture. A mechanistically rigorous distinction is needed for both interpretive coherence and risk-tiered regulation.

#### 4.1.1. Uptake Routes Diverge with Size

MPs (1 µm–5 mm) are typically ingested whole and confined to the gut lumen. Their effects are mediated by physical abrasion, gut residence time, microbiome disturbance, and additive leaching, with limited and shape-dependent translocation across the gut epithelium [71,72,98]. NPs (<1 µm) are taken up not only via gut ingestion but also through gill epithelia, integumentary surfaces, and cellular endocytic machinery. Particles below 100 nm can cross membranes directly and reach internal tissues [36,37,94]. This shift from compartmentalized luminal exposure to systemic intracellular distribution is the principal mechanistic basis for the disproportionate potency of NPs.

#### 4.1.2. Intracellular Localization Differs Accordingly

MPs that translocate are usually sequestered in phagolysosomal vesicles, where they elicit chronic lysosomal stress and low-grade inflammatory signaling. NPs can reach mitochondria, the ER, and even nuclear compartments [92,99,100]. They thereby position themselves at the very organelles whose dysfunction triggers ROS amplification, unfolded protein response (UPR) activation, and apoptotic decision-making. Direct mitochondrial association is mechanistically pivotal: it bypasses the need for diffusible signal transduction, and the particle itself becomes a localized source of dysfunction.

#### 4.1.3. Toxic Potency Is Consistently Size-Shifted

On a mass basis, NPs typically elicit cytotoxic and genotoxic responses at concentrations one to two orders of magnitude lower than equivalent mass MP doses [101,102,103]. On a particle number basis, the disparity narrows but does not disappear. This points to surface area and reactivity-related factors (not merely particle count) as drivers of the differential. Surface charge can either widen or narrow the gap. Cationic NPs are markedly more lytic than neutral or anionic NPs of the same size, sometimes producing acute hemocyte mortality at concentrations where neutral NPs are inert [49,74].

#### 4.1.4. Threshold and Dose–Response Architectures Differ

MP-induced effects are often described by relatively shallow, monotonic dose–response curves dominated by gut mechanical and microbiome-mediated processes, with substantial inter-individual variability and frequent absence of low-dose effects [63,104]. NPs frequently exhibit steeper, biphasic, or even hormetic patterns [105]. These distinct shapes point to separate mechanistic regimes operating at low (subcellular signaling) versus high (overt cytotoxicity) concentrations. Threshold concentrations for NP-induced apoptosis and DNA damage typically fall in the µg L^−1^ to low-mg L^−1^ range, whereas MP equivalents often require hundreds of mg L^−1^ to elicit comparable molecular responses [103,106,107].

#### 4.1.5. In Summary

MPs act predominantly as a chronic luminal stressor, whose toxicity is filtered through the gut epithelium, microbiota, and slow additive leaching. NPs act as a systemic intracellular stressor, whose toxicity is mediated by direct organelle interaction, reactive surface chemistry, and corona-modulated cellular trafficking. Treating NPs and MPs as a single “plastic particle” category obscures these differences and impedes mechanistic interpretation as well as risk-tiered regulation.

## 5. Cytotoxic Effects of Nano- and Microplastics

Cytotoxicity manifests across several interrelated dimensions: membrane integrity loss, mitotic disruption, programmed cell death, autophagy, and cell-cycle dysregulation. NP-induced cytotoxicity is generally more pronounced than that of MPs at equivalent mass concentrations. The pattern reflects greater number concentration, surface reactivity, and intracellular accessibility of nano-sized particles [101,102].

Membrane damage is a recurrent early event. Cationic PS nanoparticles disrupt phospholipid bilayer integrity through electrostatic interactions. The result is elevated lactate dehydrogenase (LDH) leakage, increased membrane permeability, and altered fluidity in mollusk hemocytes [108], and severe embryotoxicity consistent with cationic particle-induced membrane disruption in sea urchin (*Paracentrotus lividus*) embryos [109]. Anionic and neutral particles are less acutely lytic but can still compromise membrane function via lipid peroxidation, especially under sustained oxidative stress [62].

Cytotoxic and chronic-toxicity responses, including reduced viability and impaired physiological performance, have been reported in the freshwater crustacean *Daphnia magna* (including under nickel–microplastic co-exposure) and in the freshwater bivalve *Dreissena polymorpha* exposed to microplastic particles [110,111]. Direct cytogenetic evidence for mitotic apparatus interference (for example, spindle disruption or chromosomal mis-segregation) in invertebrates remains limited and is not established by these two studies; chromosomal and mitotic endpoints, together with their supporting evidence, are treated in Section 6.2. Cell-cycle perturbation and DNA damage response signaling have so far been characterized mainly in vertebrate models: nanoplastic exposure impaired mitochondrial energy production in developing zebrafish [112] and induced oxidative DNA damage in zebrafish embryo brain tissue [113]. These are vertebrate findings; directly comparable cell-cycle checkpoint data in invertebrate hemocytes (for example, G1/S and G2/M arrest accompanied by induction of p21 and p53 orthologs) remain scarce and represent a research gap rather than an established invertebrate response.

Apoptosis is a central cytotoxic outcome, executed through both intrinsic mitochondrial and extrinsic death receptor pathways. Mitochondrial membrane potential collapse, cytochrome c release, and caspase 3/9 activation have been documented in *M. galloprovincialis* hemocytes, with related apoptotic and immunotoxic responses reported in other marine bivalves [49,114,115]. Bax/Bcl-2 transcript imbalances and elevated TUNEL-positive nuclei in mussels are consistent with canonical apoptotic engagement [52]. Autophagy is engaged in parallel, acting as a cytoprotective mechanism but turning cytotoxic when dysregulated; autophagy-related and immune/oxidative stress responses (including changes in autophagy markers such as LC3-II/LC3-I, p62, Atg5, Atg7, and Beclin-1, where assayed) have been reported in the mussel *M. galloprovincialis* [116] and in the crustacean *Eriocheir sinensis* [117], although a fully resolved autophagic flux marker panel remains to be systematically quantified in these taxa. Necrosis, with inflammatory release of cellular contents, appears at higher exposure concentrations and with weathered or co-contaminated particles [118]. The organelle-level mechanisms underpinning these endpoints (mitochondrial–lysosomal feedback and ER stress) are integrated in Section 12.4.

A consistent pattern across studies is the dose and size dependence of cytotoxic responses. NPs elicit effects at concentrations one to two orders of magnitude lower than MPs on a mass basis [103]. Cross-study comparison is complicated by differences in particle characterization, dispersion stability, and exposure media. The need for standardized protocols is taken up in Section 14.

Taxon comparison (cytotoxicity). Cytotoxic injury is most consistently and acutely documented in filter-feeding marine bivalves (notably *Mytilus* hemocytes), in which cationic nanoplastics elicit pronounced membrane destabilization and apoptotic engagement. Crustaceans such as *Daphnia* show measurable but generally less acute single-cell cytotoxicity, with effects weighted toward reproductive and metabolic readouts, whereas nematodes (*C. elegans*) and earthworms (*Eisenia* spp.) are comparatively under-characterized at the single-cell level, with available endpoints weighted toward intestinal, coelomocyte, and whole-organism responses. These contrasts reflect different exposure routes and cell-type accessibility rather than a single shared cytotoxic mechanism, and caution against extrapolating bivalve hemocyte responses directly to other phyla.

## 6. Genotoxic Effects of Nano- and Microplastics

To consolidate the treatment of DNA damage in response to a reviewer comment, this section now serves as the central account of genotoxic endpoints. Cell-cycle and mitotic observations introduced in Section 5 are explicitly cross-referenced here (Section 6.2), and oxidative DNA lesions are revisited only in their mechanistic, MIE-anchored context in Section 7 and Section 12.3 to avoid duplication. Genotoxicity is one of the most extensively investigated outcomes of NP/MP exposure, with implications for individual fitness and, potentially, although not yet quantitatively validated, for population-level processes. The single-cell gel electrophoresis (comet) assay has shown elevated tail moment, intensity, and length in *Mytilus* and *Scrobicularia plana* tissues exposed to microplastics [119,120]. Beyond MP-specific evidence, supporting datasets from related particle and complex-contaminant exposures contribute to the broader case for invertebrate genotoxicity: fullerene C60 nanoparticles induce autophagy-linked cellular alterations in *Mytilus* digestive gland [121], and stress-response transcriptional profiling in *Eisenia andrei* from a contaminated soil reveals broad DNA damage and detoxification gene induction [122]. Direct comet assay datasets specifically for *Daphnia* and *C. elegans* under NP/MP exposure remain comparatively scarce and represent a methodological gap. Responses are typically dose-dependent and more pronounced for nano-sized and aged particles, marking DNA strand breakage as a sensitive and reproducible endpoint.

### 6.1. Resolving Subtypes of DNA Damage

Specific subtypes of DNA damage have been resolved using modified comet protocols. The alkaline comet assay detects mainly single-strand breaks (SSBs) and alkali-labile sites; neutral conditions reveal double-strand breaks (DSBs) [123]. Lesion-specific endonucleases such as formamidopyrimidine DNA glycosylase (Fpg) and endonuclease III have shown that NP exposure generates oxidative DNA lesions, including 8-oxo-7,8-dihydro-2′-deoxyguanosine (8-oxodG), a hallmark of ROS-mediated genotoxicity [124,125]. Elevated γ-H2AX foci, indicative of DSBs, have been reported in invertebrate cell lines and primary hemocytes exposed to NPs. The pattern is consistent with DSB formation as a downstream consequence of replication fork collapse at unrepaired SSBs and oxidative lesions [126].

### 6.2. Cytogenetic and Chromosomal Endpoints

Cytogenetic endpoints reinforce the genotoxic burden of plastic particles. Micronucleus frequency is a robust integrative biomarker that reflects both chromosomal breakage (clastogenicity) and whole chromosome loss (aneugenicity). It is elevated in mussel hemocytes and earthworm coelomocytes exposed to PS, PE, and PVC particles [51,61]. Nuclear abnormalities (binucleated cells, lobed nuclei, blebbed nuclei, and nuclear buds) rise dose dependently [127]. Chromosomal aberrations have been documented in *Chironomus riparius* salivary gland polytene chromosomes, manifesting as breaks, asynapsis, and ectopic pairing [55], although such cytogenetic analyses remain less common in invertebrate NP/MP work than the comet and micronucleus assays. Sister chromatid exchange (SCE), which reflects homologous recombination during S-phase repair, is markedly underexplored in invertebrate NP/MP toxicology. Given the strong replication stress signatures reported in transcriptomic studies, SCE assays would offer mechanistically valuable evidence on homologous recombination-mediated repair currently inferred only indirectly. It should be emphasized, however, that the scarcity of SCE data is not solely a conceptual omission but also reflects a genuine technical constraint. With the notable exception of *Chironomus*, whose large polytene chromosomes are comparatively favorable for cytogenetic scoring, most standard invertebrate ecotoxicological models have small, numerous, or poorly spread chromosomes that make reliable SCE differential staining and scoring technically difficult. Closing this gap will therefore require methodological development (for example, optimized metaphase preparation and BrdU-substitution protocols) as much as a shift in research priorities.

### 6.3. Replication Stress as an Emerging Genotoxic Mechanism

Replication stress (the slowing or stalling of DNA replication forks) is increasingly recognized in vertebrate cell systems as a consequence of chronic oxidative and chemical insult, and is being considered as a candidate mechanism in invertebrate NP/MP toxicology, although direct experimental confirmation in invertebrate models remains limited. Oxidative DNA lesions, ribonucleotide misincorporation under perturbed dNTP pools, and DNA–protein crosslink formation can all impede replisome progression in principle, leading to fork stalling, single-stranded DNA accumulation, and ATR–Chk1 pathway activation [126,128,129]. Indirect signatures consistent with replication stress have been reported in invertebrate transcriptomic datasets, in which NP exposure was associated with the upregulation of replication-associated checkpoint kinases and homologous recombination components such as RAD51 and BRCA1 orthologs [43,106,130,131]. Whether these transcriptional changes reflect bona fide replication fork stress, generalized DNA damage response engagement, or transcriptional bystander effects of redox imbalance has not been resolved in invertebrate models. Persistent replication stress is, in better-characterized systems, sufficient to drive double-strand break formation through fork collapse and to contribute to genomic instability [128,132]. Including replication stress-specific markers (phospho-Chk1, RPA32 phosphorylation, ssDNA accumulation) in invertebrate genotoxicity batteries would help to determine whether the mechanism inferred from transcriptional signatures is operative at the protein and lesion level [133].

### 6.4. DNA Repair Pathway Disruption

Disruption of DNA repair pathways is increasingly recognized as a mechanistic amplifier of genotoxicity. Transcriptomic and qPCR analyses have shown altered expression of base excision repair (ogg1, ape1), nucleotide excision repair (xpa, xpc), mismatch repair (msh2, mlh1), and double-strand break repair (rad51, brca1-like) genes in *D. magna*, *C. elegans*, and *M. galloprovincialis* exposed to PS-NPs [106,130,131]. Such dysregulation can compromise genome maintenance and allow unrepaired lesions and mutations to propagate to daughter cells.

### 6.5. Underexplored Endpoints: Telomere Damage, DNA–Protein Crosslinks, and Beyond

Telomere dynamics under plastic particle exposure have only recently begun to receive attention in invertebrate ecotoxicology, and the available evidence base is correspondingly thin. Shortened telomere length and altered shelterin component expression have been reported in long-term NP-exposed mussels and in *C. elegans* [59]; recent work in other aquatic models has further explored the relationship between MP exposure and telomere dynamics, with mixed outcomes [134]. These observations rest on a small number of studies and have not yet been independently replicated across laboratories. The mechanistic chain that has been proposed—oxidative damage at telomeric guanine triplets, inhibition of telomerase activity under chronic stress, and downstream cellular senescence and reproductive aging—is biologically plausible and consistent with parallel findings in mammalian models, but the causal sequence has not been formally established in invertebrate models. Reduced regenerative capacity and reduced fitness should therefore be treated as a hypothesis that motivates further investigation rather than as a mechanism documented at the level of evidence available for, say, oxidative DNA damage. DNA–protein crosslinks (DPCs) are another largely unexplored endpoint in invertebrate plastic ecotoxicology. They are covalent adducts between proteins and DNA that can in principle be induced by ROS, aldehydes, or reactive electrophiles released from weathered plastics. DPCs uniquely impede both transcription and replication: replication-coupled repair proceeds through SPRTN-mediated proteolysis and the 26S proteasome, while transcription-blocking DPCs are processed by CSB- and CRL4CSA-coupled repair pathways [135,136]. Both routes are metabolically costly and, when overwhelmed, contribute to fork collapse and persistent transcriptional silencing. To our knowledge, systematic DPC quantification has not yet been reported for NP/MP-exposed invertebrates. This is a clear methodological gap, but it is one that must be approached with analytical caution rather than by routine application of KCl–SDS precipitation alone. Organisms exposed in sediment, soil, or organic-rich water take up substantial quantities of natural aldehydes and humic substances, which elevate and add variability to baseline DPC levels; distinguishing a genuinely plastic-specific DPC signature from this fluctuating environmental background is therefore a difficult bioanalytical problem. Reliable measurement will require carefully matched unexposed and field-reference controls, leachate and solvent controls, and ideally orthogonal confirmation (for example, mass-spectrometric crosslink identification) rather than KCl–SDS precipitation or immunoblotting in isolation.

### 6.6. From Molecular Damage to Evolutionary Consequences

Translating molecular genotoxicity into population-level outcomes requires consideration of mutation accumulation, selection, and demographic processes. These are at least in principle tractable in short-generation invertebrate models, but they have not been quantitatively measured under NP/MP exposure in any system to date. The inferences drawn here should therefore be read as hypothesis-generating rather than demonstrated outcomes. On theoretical grounds, persistently elevated DNA damage combined with compromised repair capacity is expected to increase mutation load over time, particularly in germline tissues, where DNA repair fidelity directly influences offspring genotype. In clonal reproducers such as *Daphnia*, somatic and germline mutations cannot be purged by recombination, so chronic NP/MP exposure could in principle contribute to genetic erosion under sustained selective pressure. This is mechanistically plausible, but direct evidence in invertebrate plastic ecotoxicology is currently absent; mutation accumulation designs with sequencing-based mutation calling would be required to validate it. In sexually reproducing taxa, even modest increases in deleterious mutation rate could in principle compound across generations and depress mean fitness, although the relevant population-genetic parameters (per-generation mutation rate increment, effective population size, selection coefficient) remain unmeasured for plastic exposures. Replication stress and telomere attrition contribute, in better-characterized systems, to senescence-like phenotypes that depress reproductive output; whether this translates into increased extinction risk in small, fragmented invertebrate populations remains mechanistically plausible but currently indirect. The conceptual chain DNA damage → repair failure → mutation accumulation → fitness depression provides a principled framework that treats molecular genotoxicity as a potentially evolutionarily relevant outcome rather than a purely endpoint-level observation. Integrating emerging endpoints (replication stress markers, telomere dynamics, DPCs, mutation accumulation rates) into standard genotoxicity batteries will be important. Whether the resulting readouts ultimately support quantitative predictions at the population or community scale remains a research priority rather than a present capability.

## 7. Oxidative Stress and Cellular Damage

Oxidative stress is the most consistently reported response to NP/MP exposure across invertebrate models and occupies a mechanistically central role in cytotoxicity, genotoxicity, and molecular reprogramming. Particle interaction with cellular surfaces, mitochondria, and lysosomes generates excessive ROS (superoxide anion (O_2_•^−^), hydrogen peroxide (H_2_O_2_), and hydroxyl radical (•OH)) that overwhelm antioxidant defenses and produce macromolecular oxidative injury [137,138]. Whether ROS functions as a primary initiator or a downstream amplifier depends on particle physicochemistry, exposure regime, and tissue context, an interpretive question taken up critically in the section Is Oxidative Stress a Primary Driver or a Secondary Amplifier? A Critical Appraisal.

ROS overproduction has been documented in *D. magna*, *Artemia franciscana*, *C. elegans*, *Eisenia* spp., and various mollusks following exposure to PS, PE, and PVC particles, with NPs typically eliciting greater responses than MPs [58,139,140]. The relevant ROS sources (particle surface redox chemistry, mitochondrial electron transport dysfunction, and immune-cell NADPH oxidase activation) are dissected in their MIE-anchored form in Section 12.3; the central observation at this level is that these sources are not mutually exclusive and their combined output overwhelms antioxidant defenses, producing macromolecular oxidative injury [141,142].

Lipid peroxidation, quantified through malondialdehyde (MDA) and 4-hydroxynonenal (4-HNE) accumulation, is a near-universal finding in NP/MP-exposed invertebrates. It reflects the susceptibility of polyunsaturated membrane lipids to ROS-mediated oxidation [53,143]. The ensuing compromise of membrane fluidity and integrity feeds back onto ion homeostasis, mitochondrial function, and signal transduction. Protein oxidation, evidenced by elevated protein carbonyl content and altered thiol status, is mechanistically linked to inactivation of structural and enzymatic proteins, including those involved in DNA repair and antioxidant defense [144].

The antioxidant defense network responds dynamically. Superoxide dismutase (SOD), catalase (CAT), glutathione peroxidase (GPx), and glutathione S-transferase (GST) typically show biphasic responses. Initial induction reflects compensatory upregulation. Inhibition follows under prolonged or high-dose exposure as adaptive transcriptional capacity is overwhelmed and oxidative burden inactivates the enzymes themselves [145,146]. The reduced-to-oxidized glutathione ratio (GSH/GSSG) is a particularly sensitive index of cellular redox state, declining under sustained NP/MP exposure and correlating with elevated oxidative DNA damage [147]. The Nrf2-Keap1 pathway, conserved across invertebrates and central to the transcriptional regulation of antioxidant and detoxification genes, has been shown to be activated in *C. elegans* (SKN-1 ortholog) and *Mytilus* hemocytes exposed to NPs, providing a molecular basis for adaptive responses [148,149]. Table 2 (presented in Section 13 alongside Table 1) consolidates the antioxidant and detoxification genes discussed here together with the principal stress, repair, apoptotic, immune, reproductive, and epigenetic genes reported across invertebrate models, to assist the selection of physiological and molecular markers for future studies.

### Is Oxidative Stress a Primary Driver or a Secondary Amplifier? A Critical Appraisal

The near-ubiquity of oxidative stress reports in NP/MP studies has often been read as evidence that ROS is the primary driver of plastic particle toxicity. A more careful examination of the available data suggests instead that oxidative stress occupies a dual position (a proximate molecular initiating event in some exposure regimes, a downstream amplifier in others) and that conflating these roles obscures rather than clarifies causal interpretation.

Several observations are consistent with a primary role. Weathered plastics bearing carbonyl, peroxide, and quinone-like surface groups can catalyze redox reactions on direct contact with biological media, generating ROS prior to any cellular signaling event [78,80,141]; cationic NPs that disrupt mitochondrial membranes have likewise been associated with immediate, particle-localized ROS bursts that appear to precede transcriptional responses [49,99,154]. In these contexts, the particle itself behaves as a ROS source, and oxidative damage may be the mechanistic origin of downstream cytotoxic and genotoxic effects.

Other lines of evidence are equally consistent with a secondary role. Lysosomal destabilization, ER stress, and inflammatory signaling all generate substantial ROS as downstream amplifiers rather than initiators [91,98,155]. Pristine, charge-neutral PS nanoparticles in several studies induced ROS only after intracellular accumulation and organelle interaction, suggesting that uptake and trafficking can precede ROS generation [106,117]. Antioxidant rescue experiments yield inconsistent results across the literature: in some studies, N-acetylcysteine or glutathione supplementation attenuated NP-induced apoptosis, supporting causal primacy of ROS [58,117]; in others, antioxidant pretreatment failed to rescue downstream phenotypes, suggesting that ROS-independent pathways (direct membrane disruption, mechanical injury, additive-mediated endocrine effects) make substantial parallel contributions [110,156].

A more defensible conceptualization, and the one adopted here, is that oxidative stress is best treated as an obligate node within a multi-input, multi-output mechanistic network rather than a unitary causal driver [157]. Whether ROS is upstream or downstream appears to depend on particle physicochemistry (weathered vs. pristine; cationic vs. neutral), exposure regime (acute vs. chronic), and tissue context (mitochondrion-rich vs. epithelial). Apparent inconsistencies across studies, induction vs. inhibition of antioxidant enzymes, and antioxidant rescue vs. no rescue, are likely to reflect differences in these parameters more than genuine biological discordance. The methodological implication is that progress will depend on time-resolved, mechanistically informed experimental designs that explicitly resolve the temporal sequence of ROS generation relative to other early key events.

## 8. Molecular and Epigenetic Responses

Molecular responses to NP/MP exposure encompass coordinated transcriptional reprogramming of stress, repair, apoptotic, and inflammatory pathways. Epigenetic remodeling is increasingly recognized as a parallel and persistent layer. Transcriptomic analyses across invertebrate models reveal consistent up- or downregulation of genes encoding heat-shock proteins (*hsp70*, *hsp90*), metallothioneins, cytochrome P450 enzymes, glutathione-related enzymes, DNA repair components, and pro- and anti-apoptotic regulators [158,159,160]. In *D. magna*, PS-NPs elicit differential expression of vitellogenin, ecdysone-related receptors, and chitin-metabolism genes, mechanistically linking molecular perturbation to reproductive and developmental outcomes [150].

Inflammatory and immune-related responses are widely reported across invertebrate models after NP/MP exposure. In mollusk hemocytes, this includes dysregulation of lysozyme, antimicrobial peptides, Toll-like receptor pathway components, and NF-κB orthologs, reflecting activation of innate immune surveillance and chronic low-grade inflammation [32,50]. In soil invertebrates such as earthworms, comparable molecular-level immune and stress-response perturbations have been documented at the transcriptional level, although a fully resolved immune gene panel comparable to that available for mollusks remains to be established [18,122]. The downstream oxidative-inflammatory amplification loop is described in Section 12.5.

Epigenetic mechanisms are emerging as critical, yet underappreciated, dimensions of NP/MP toxicity. Altered global DNA methylation, assessed via 5-methylcytosine immunostaining or LC-MS, has been reported in *D. magna* and *M. galloprovincialis* exposed to NPs. Both hyper- and hypomethylation have been observed, with the direction depending on particle type and exposure duration [43,161,162]. The available invertebrate datasets remain limited and heterogeneous: the better-characterized examples involve sub-100-nm polystyrene nanoparticles at sub-mg/L to low-mg/L concentrations (for example, 75-nm PS nanoparticles at 0.1–1 mg/L in *Daphnia* [43]), with affected loci concentrated in the promoters of antioxidant, immune, heat-shock, and developmental genes; the mechanistic basis for the opposing global directions (acute high-dose, locus-specific hypermethylation versus chronic low-dose, global hypomethylation linked to one-carbon-pathway depletion) is examined in Section 14.1.2. Fully locus-resolved, dose- and size-matched methylation maps are nonetheless still scarce. Locus-specific methylation changes affect promoters of stress response, immune, and developmental genes, providing a molecular substrate for persistent transcriptional reprogramming. Histone post-translational modifications, including altered H3K4me3 and H3K9me3 marks, have been documented in *C. elegans* exposed to PS-NPs, linking exposure to chromatin-level remodeling [163].

MicroRNA (miRNA) regulation adds a further layer of post-transcriptional control. Differential expression of conserved miRNAs targeting apoptosis, immune, and stress-response pathways has been reported in mollusks and *Daphnia* exposed to NPs/MPs, suggesting that miRNA-mediated regulation may contribute to both adaptive and maladaptive responses [151,152,153]. The heritability of epigenetic marks across generations, particularly in clonal *Daphnia* lineages, has been documented for several environmental stressors and is consistent with the possibility that NP/MP exposure can imprint persistent molecular signatures with downstream consequences for population structure [164,165]. We caution, however, that direct evidence linking specific NP/MP-induced epigenetic marks to heritable phenotypic change in invertebrates remains limited; the population- and biodiversity-level inferences that follow from this premise should be regarded as motivated hypotheses rather than established outcomes.

## 9. Multi-Omics Approaches

The integration of transcriptomic, proteomic, metabolomic, and epigenomic data is gradually reshaping NP/MP ecotoxicology and shifting the field from descriptive endpoint cataloging toward mechanistic systems toxicology. Transcriptomic and broader omics profiling in NP/MP-exposed *Daphnia magna* and marine bivalves, with complementary evidence from vertebrate models such as zebrafish, has revealed coordinated dysregulation of pathways related to oxidative stress, lipid metabolism, energy production, immunity, and proteostasis [60,166,167,168]. Proteomic analyses corroborate and extend these findings, identifying altered abundance of cytoskeletal, mitochondrial, ribosomal, and detoxification proteins, frequently with post-translational modifications consistent with oxidative or carbonyl stress [169,170,171].

Metabolomic studies have begun to illuminate the downstream physiological consequences. Reported signatures include disrupted amino acid metabolism, perturbed tricarboxylic acid (TCA) cycle intermediates, altered lipid and energy metabolism, and dysregulated osmolyte profiles in NP/MP-exposed *Daphnia*, mussels, and earthworms [172,173]. These metabolic patterns provide functional readouts of cellular stress and offer candidate biomarkers for environmental monitoring, with the caveat that metabolome-level signatures integrate over many upstream perturbations and rarely identify a unique mechanistic source. Epigenomic approaches (reduced representation bisulfite sequencing (RRBS) and ChIP-seq) are beginning to map genome-wide methylation and chromatin landscapes in NP-exposed invertebrates, although such studies are still in their infancy and the inferences they support remain preliminary [174,175].

**Figure 3:**
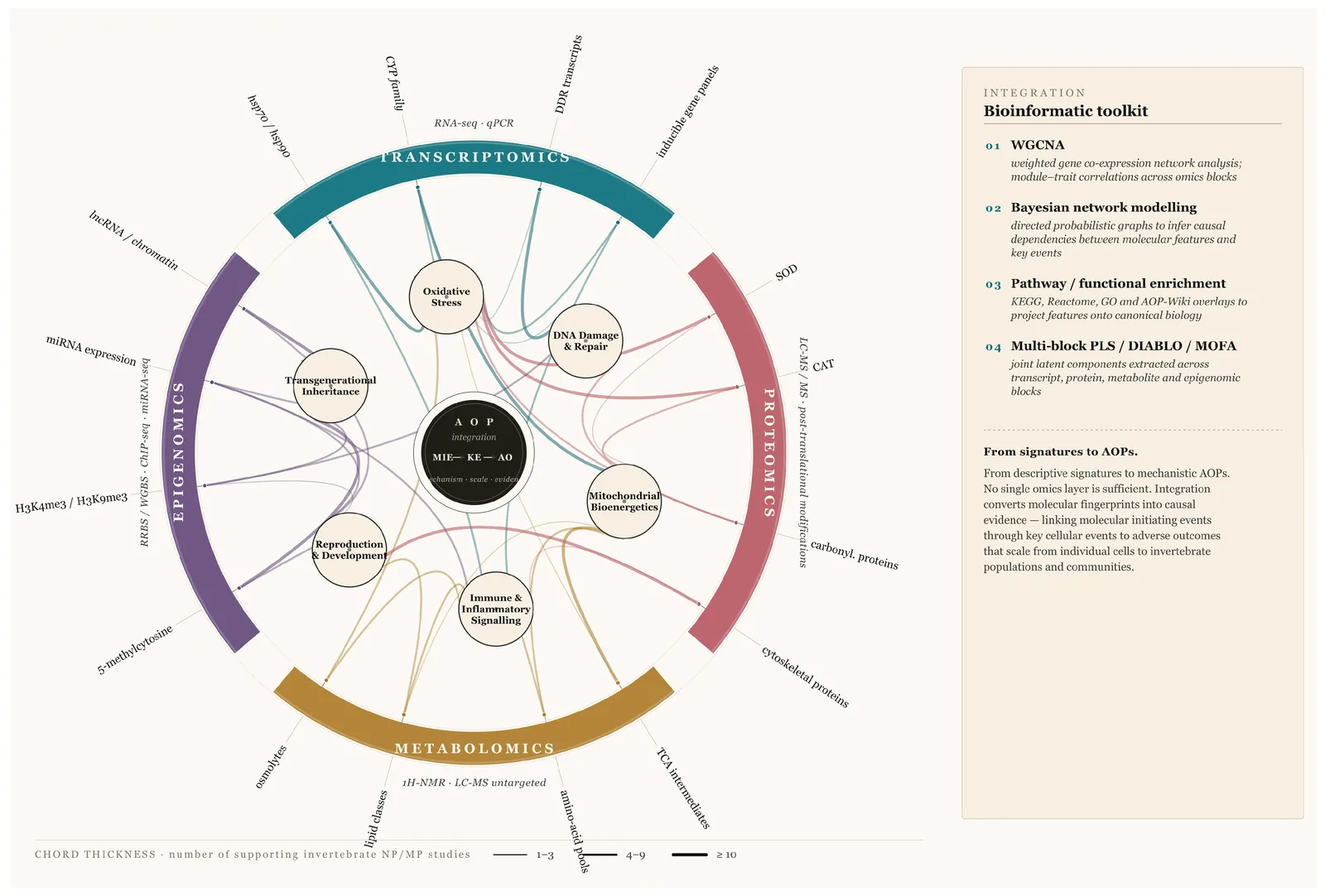
Multi-omics integration map for NP/MP toxicity in invertebrates. Outer ring: the four omics layers and their representative readouts, transcriptomics (RNA-seq, qPCR; *hsp70*/90, CYP genes, DDR transcripts), proteomics (LC-MS/MS, post-translational modifications; SOD, CAT, carbonylated and ubiquitinated proteins, cytoskeletal proteins), metabolomics (1H-NMR, untargeted LC-MS; TCA-cycle intermediates, amino acid pools, lipid classes, osmolytes), and epigenomics (RRBS/WGBS, ChIP-seq, miRNA-seq; 5-methylcytosine patterns, H3K4me3/H3K9me3, miRNA expression). Inner chords link omics features to six AOP-relevant biological themes (oxidative stress, DNA damage and repair, mitochondrial bioenergetics, immune and inflammatory signaling, reproduction and development, and transgenerational inheritance), with chord thickness indicating, qualitatively, the relative emphasis a given omics–theme link receives in the synthesized invertebrate literature. Chord widths are schematic and are not derived from a systematic, reproducible count of studies and should not be read quantitatively. The transgenerational inheritance theme in particular is included as an emerging, hypothesis-level layer that is well supported only in *C. elegans* and remains largely unvalidated in macroinvertebrates. The central node represents AOP integration (MIE → key events → adverse outcomes). The side panel lists indicative bioinformatic integration approaches (WGCNA, Bayesian network modeling, pathway enrichment, multi-block PLS/DIABLO/MOFA). The figure is intended as a conceptual synthesis: no single omics layer is sufficient on its own, and integrative analysis is what converts molecular signatures into AOP-grade evidence.

Genuine multi-omics integration (the joint analysis of two or more omics layers within a single experimental framework) remains rare in invertebrate NP/MP work, but is uniquely informative where it has been attempted. Integrative analyses can identify cross-layer concordance, help distinguish causal from correlative perturbations, and link molecular initiating events to downstream key events within an AOP framework [176]. Bayesian network modeling, weighted gene co-expression network analysis (WGCNA), and multi-block latent-variable approaches are increasingly used to extract systems-level patterns from such datasets [177,178]. Recent transcriptomic–metabolomic integrations in invertebrate NP exposure studies have shown that energy metabolism reprogramming and one-carbon-pathway perturbation are recurrent multi-omics signatures. These observations are consistent with the possibility that some of the methylation changes reported in NP-exposed invertebrates reflect upstream depletion of S-adenosylmethionine pools rather than direct effects on DNA methyltransferase activity [168,175]; this remains a hypothesis to be tested through targeted one-carbon flux measurements rather than a settled mechanism (Figure 3). 

## 10. Reproductive and Developmental Effects

Reproductive impairment and developmental abnormalities are among the most ecologically relevant outcomes of NP/MP exposure. They translate molecular and cellular injury directly into population-level consequences and have clear implications for invertebrate biodiversity. In *D. magna*, chronic exposure to PS-NPs reduces fecundity, prolongs the time to first brood, decreases neonate body size, and increases the incidence of malformed and aborted embryos [39,107]. These effects have been linked mechanistically to disrupted vitellogenesis, altered ecdysone signaling, oxidative stress in the ovary, and compromised energy allocation [40].

In *C. elegans*, NP exposure reduces brood size, delays egg-laying, and induces germline apoptosis, with concomitant upregulation of *ced-3*, *ced-4*, and egl-1 and altered insulin/IGF-1 signaling [47,48].

Developmental endpoints in *Chironomus* spp. and *Artemia* spp. include altered hatching rates, delayed metamorphosis, mouthpart deformities, and reduced larval survival under NP/MP exposure, with effects most pronounced for weathered particles and additive-bearing matrices [56,57]. Taken together, these findings support the use of reproductive and developmental toxicity as integrative endpoints that capture the cumulative impact of molecular, cellular, and physiological perturbations on individual fitness. The extension from individual-fitness reductions in laboratory exposures to consequences for recruitment and persistence in natural invertebrate populations is mechanistically plausible but currently indirect, and depends on population-level demographic data that are seldom collected alongside the toxicological endpoints reviewed here.

## 11. Multigenerational and Transgenerational Effects

Multigenerational and transgenerational toxicology has emerged as a critical frontier in NP/MP research. The central question is whether exposure-induced phenotypes persist beyond the directly exposed generation. Multigenerational effects describe responses observed in offspring whose germline was exposed in utero or in ovo (F0–F1, sometimes F2). Transgenerational effects, strictly defined, require persistence into generations not directly exposed (F3 in sexually reproducing organisms; later generations in clonal species) [179].

Studies in *D. magna* have shown that maternal NP exposure reduces fitness in subsequent generations, even when offspring are reared in clean media. Effects on reproduction, body size, and stress tolerance have been documented across F1–F3 [41,42]. Clonal reproduction in *Daphnia* makes the dissection of epigenetic inheritance particularly tractable, and altered DNA methylation and miRNA profiles have been implicated as molecular carriers of transgenerational effects [180,181]. In *C. elegans*, a powerful model for transgenerational epigenetics, NP exposure induces phenotypes that persist across multiple generations, accompanied by altered histone marks and small RNA pathways [46]. A particularly informative study extended the design to five generations and demonstrated that a single maternal PS-NP exposure causes reproductive decline through F4, accompanied by hypomethylation of the *ced-3* promoter, and that RNAi of the methylation-related factors *met-2*, *set-2*, and *spr-5* abolished the transgenerational effect [182], providing one of the few invertebrate datasets in which an epigenetic mechanism has been causally interrogated rather than merely correlated. Complementary work at predicted environmental concentrations (1–10 µg L^−1^) further shows transgenerational toxicity transduced through germline nuclear hormone receptor and Ephrin-signaling pathways [183], suggesting that the phenotypes observed at higher experimental doses are not artifacts of overload.

Marine bivalves have received less attention in this context. In *Crassostrea gigas*, early-life-stage exposure to common environmental contaminants and intergenerational exposure to environmentally-aged microplastics have both been shown to compromise embryonic and larval performance, indicating susceptibility windows that may also be relevant for NP/MP risk in bivalves more broadly [184,185]. These bivalve studies, however, examine directly or germline-exposed parental and offspring generations and therefore constitute multigenerational, not transgenerational, evidence; stable transmission to unexposed generations has not been demonstrated in this taxon, and the corresponding evidence strength is best regarded as emerging rather than moderate. Mechanistic understanding of transgenerational inheritance in invertebrate plastic ecotoxicology is still incomplete. The convergence of epigenetic remodeling, germline oxidative damage, and altered parental provisioning provides a mechanistically plausible framework, but the relative contributions of these processes have not been disentangled in NP/MP exposures. With the partial exception of work in *C. elegans* that has now reached F4–F5 [46,182], most published “transgenerational” experiments examine only one or two filial generations and therefore strictly speaking address multigenerational rather than transgenerational inheritance. Whether persistent multigenerational effects, where they occur, scale up to influence long-term population trajectories or invertebrate community structure under chronic environmental contamination remains an open empirical question. We frame this as a hypothesis-generating proposition that warrants systematic investigation across taxonomic groups and reproductive modes, with extended generational designs and matched ecological monitoring, rather than as a documented outcome.

## 12. Mechanistic Integration: From Primary Molecular Initiating Events to Adverse Outcomes

### 12.1. Evidence-Strength Annotation

To strengthen the AOP architecture and to make evidence appraisal transparent rather than impressionistic, each pathway component below is annotated using a three-tier qualitative convention adapted from quantitative AOP practice. The grading is not a numerical score and does not substitute for formal risk-of-bias assessment. It is a structured summary judgment that integrates four criteria, applied uniformly across MIEs, key events, and adverse outcomes.

(i) Mechanistic support—the extent to which the proposed causal link between the pathway component and its upstream/downstream neighbors is anchored in defined molecular, cellular, or physiological processes rather than inferred from correlative endpoint readouts;
(ii) Reproducibility—whether the effect has been reported by independent laboratories, with different particle batches and exposure protocols, yielding broadly concordant directional outcomes;
(iii) Taxonomic breadth—whether the effect has been observed across multiple invertebrate taxa spanning different phyla and ecological niches, or remains confined to a single model system;
(iv) Causal validation—whether the mechanistic claim has been tested by pharmacological inhibition, genetic perturbation (knockout, knockdown, CRISPR), antioxidant or chelator rescue, or other interventional designs that move beyond observational association.

Tier assignment follows a conservative rule. A component is annotated as strong only when all four criteria are satisfied: mechanistic support is explicit, reproducibility is demonstrated across independent groups, taxonomic breadth covers at least two phyla, and causal validation has been achieved through interventional or rescue experiments. It is annotated as moderate when mechanistic support and reproducibility are established, but either taxonomic breadth is limited (one phylum or a small number of model species) or causal validation rests on association rather than perturbation. It is annotated as emerging when mechanistic support is plausible, and one or two studies report concordant findings, but reproducibility across independent laboratories, taxonomic breadth, and causal validation are largely absent; the mechanism is often inferred from related (mammalian, vertebrate, or in vitro) systems. Borderline cases were resolved by joint consensus between the two authors (see Section 2.5), with the higher tier assigned only when all four criteria were defensible. Where the two authors disagreed on tier assignment, the lower tier was used. Together, the four-criterion framework and the conservative-assignment rule are intended to make the grading systematic and reproducible, without claiming the precision of a numerical meta-analytic score.

### 12.2. Defining Primary Molecular Initiating Events for NP/MP Exposure

The adverse outcome pathway (AOP) framework requires explicit definition of the molecular initiating event (MIE), the earliest measurable interaction between a stressor and a biomolecular target that begins the causal chain leading to an adverse outcome [30]. For chemical toxicants, the MIE is typically a discrete receptor-binding or enzyme-inhibition event. For particle stressors such as NPs and MPs, the MIE is best treated not as a single event but as a defined set of physical and chemical interactions that initiate downstream key events. We propose four primary MIEs for NP/MP toxicity in invertebrates.

#### 12.2.1. MIE-1: Direct Membrane Interaction

Electrostatic and hydrophobic interactions between charged or hydrophobic particle surfaces and phospholipid bilayers, leading to membrane deformation, lipid disordering, and (particularly for cationic NPs) pore formation [37,74,109] [Evidence: strong]. Multiple independent studies in mollusk hemocytes, *Daphnia*, and *C. elegans* demonstrate dose-dependent membrane disruption with cationic PS-NH_2_ producing markedly stronger effects than anionic or neutral counterparts; charge-modulation experiments provide direct causal support.

#### 12.2.2. MIE-2: Protein Corona Formation

Adsorption of biomolecules onto particle surfaces upon contact with biological fluids, generating a dynamic corona that modulates particle recognition, dictates the uptake pathway, and can sequester or denature corona-incorporated proteins [93,94] [Evidence: strong]. The role of corona composition in dictating uptake and trafficking is well established in nanotoxicology and confirmed in invertebrate exposure media; corona-stripping and corona-engineering controls have causally linked corona identity to cellular response.

#### 12.2.3. MIE-3: Surface-Catalyzed Redox Chemistry

Generation of ROS at the particle surface through Fenton-like reactions on weathered surfaces bearing carbonyl, peroxide, or quinone-like functionalities, occurring prior to and independent of cellular signaling responses [78,80,141] [Evidence: moderate]. Cell-free assays and weathered-particle exposures support the mechanism, but disentangling surface-catalyzed ROS from organelle-derived ROS in vivo remains methodologically challenging.

#### 12.2.4. MIE-4: Trojan Horse Delivery

Transport of adsorbed co-contaminants (metals, hydrophobic organic chemicals) and leached additives to intracellular compartments at concentrations not achievable by waterborne exposure to the contaminant alone [84,85,86,142] [Evidence: moderate]. The hypothesis is supported by sorption thermodynamics and by mixture-toxicity data in mussels and *Daphnia*, but quantitative validation under environmentally realistic loadings is still incomplete and contested in some contexts [85,186]. Strictly, Trojan horse delivery is a toxicokinetic transport phenomenon rather than a molecular initiating event in the classical sense: the particle acts as a vector that raises the intracellular concentration of a secondary stressor, whereas the initiating molecular event proper is the subsequent interaction of the delivered species with its target (for example, aryl-hydrocarbon-receptor activation by PAHs, enzyme inhibition, or metal–protein binding). We therefore retain MIE-4 as a delivery step that precedes and potentiates a chemical MIE rather than as an initiating molecular interaction in its own right, and the resulting effects are correctly attributed to the delivered contaminant rather than to the plastic particle as such.

These MIEs are not mutually exclusive. For any given particle–organism combination, two or more typically operate in parallel, with relative contributions determined by polymer chemistry, surface modification, weathering state, and biological context. Distinguishing them experimentally (through corona stripping, surface functionalization, antioxidant rescue, and additive depletion controls) remains a major methodological frontier. Each MIE feeds into a partly shared, partly distinct set of key events. Direct membrane interaction (MIE-1) and Trojan horse delivery (MIE-4) preferentially initiate mitochondrial and lysosomal dysfunction. Protein corona formation (MIE-2) shapes the uptake route and thereby the intracellular compartment first exposed to the particle. Surface-catalyzed redox chemistry (MIE-3) directly seeds reactive oxygen species (ROS) generation prior to any cellular signaling response. The resulting ROS, organelle dysfunction, and downstream signaling do not, however, constitute four independent linear cascades. They form a tightly coupled network in which ROS amplification, organelle injury, inflammatory signaling, DNA damage response (DDR) engagement, and epigenetic remodeling reinforce one another. The following subsections describe this network at increasing levels of integration, beginning with ROS as the most frequently observed downstream node.

### 12.3. Reactive Oxygen Species as a Frequently Engaged but Non-Obligatory Node

ROS generation appears as a recurrent downstream node from all four MIEs in many invertebrate datasets [Evidence: strong for ROS as a downstream node; moderate for its position as bligatory central node is not supported in this review]. We stress that ROS is a frequently engaged node rather than an obligatory bottleneck through which every initiating event must pass. Several toxicity routes can operate in parallel with, or independently of, ROS, including direct mechanical injury and membrane perforation, membrane wrapping and lipid disordering, lysosomal and ER stress that can precede measurable ROS accumulation, and additive- or co-contaminant-mediated effects (for example, endocrine disruption or receptor binding by leached chemicals) that need not be redox-initiated. Forcing all MIEs through a single ROS step would therefore overstate its causal centrality and obscure these parallel modes of action; the network framing adopted here (Section 12.6) deliberately accommodates ROS-dependent and ROS-independent routes side by side. The contributing sources are mechanistically distinguishable. Surface-catalyzed Fenton-like chemistry on weathered particles (MIE-3) seeds ROS directly at the particle–biological interface. Mitochondrial electron transport dysfunction, precipitated by direct membrane interaction (MIE-1) or by Trojan horse-delivered toxicants (MIE-4), generates ROS endogenously. NADPH oxidase activation in immune cells responding to corona-decorated particles (MIE-2) contributes ROS through phagocyte effector chemistry [187]. ROS in turn oxidize lipids, proteins, and nucleic acids, producing membrane damage, enzyme inactivation, and DNA lesions, including 8-oxodG, abasic sites, and strand breaks [128]. The association between oxidative stress and genotoxicity is consistent with a causal interpretation in which ROS-induced DNA lesions, when unrepaired, contribute to mutagenesis, micronucleus formation, and chromosomal instability [188]. We note, however, that the temporal ordering required to formally establish ROS as the proximate cause (rather than a parallel or downstream marker) of these endpoints has been resolved in only a subset of invertebrate studies. Sustained ROS production also injures the organelles whose dysfunction was its initial source, generating the self-amplifying feedback examined next.

### 12.4. Organelle-Specific Key Events

Mitochondrial dysfunction is among the most consistently reported organelle-level events downstream of NP exposure [Evidence: strong]. This rating applies to exposures ≤1 g L^−1^; observations restricted to ≥1 g L^−1^ are treated as non-specific physical-overload signals (see the section Environmentally Relevant Versus Experimental Concentrations: A Critical Appraisal). NP-associated mitochondrial membrane potential collapse, disrupted electron transport, and impaired ATP production amplify ROS generation, trigger cytochrome c release, and engage intrinsic apoptosis [154,189]. Mitochondrial ROS in turn destabilize neighboring lysosomes by oxidizing lysosomal membrane lipids and triggering partial cathepsin release. Lysosomal destabilization is inferred from reduced lysosomal membrane stability assays, cathepsin redistribution, and altered autophagic flux; it disrupts cellular proteostasis and intersects with the apoptotic and autophagic pathways already activated upstream [155] [Evidence: strong]. Recent mechanistic refinement distinguishes graded “minority” lysosomal membrane permeabilization (a sublethal regime in which a small number of lysosomes release cathepsins in a spatially restricted manner) from frank lysosomal rupture; this graded view is consistent with the dose- and time-dependent cathepsin responses observed across NP/MP studies and reframes apparent inconsistencies in cathepsin-readout magnitudes between studies as differences in regime rather than as biological discordance [190] [Evidence: moderate]. Released cathepsins can in turn permeabilize the outer mitochondrial membrane, closing a self-amplifying mitochondrial–lysosomal feedback loop. This loop has been characterized in mammalian particle exposure systems and is mechanistically plausible but as yet incompletely tested in invertebrate NP/MP toxicity [191] [Evidence: emerging in invertebrate models]. A parallel organelle response is the activation of the endoplasmic reticulum unfolded protein response (UPR) under sustained exposure. UPR engagement is indicated by upregulation of *bip*, *chop*, and *xbp1* and by p38-MAPK signaling, and prolonged activation contributes to apoptosis and to amplification of inflammatory signaling [98] [Evidence: moderate]. The strongest mechanistic support comes from *C. elegans* p38-MAPK/UPR studies, with corroborating but more limited data from mussel digestive gland. ER stress thus links organelle injury to the inflammatory and DNA damage response cascades described next.

### 12.5. Inflammatory and DNA Damage Response Cascades

Two distinct but coupled response systems are engaged downstream of the organelle-level events above. Inflammatory signaling cascades mediated by NF-κB, MAPK, and Toll-like-receptor pathways respond to mitochondrial- and ER-derived stress signals (mitochondrial DNA release, ATP depletion, UPR effectors) and amplify oxidative and cellular damage through secondary ROS and reactive nitrogen species, immune-cell recruitment, and cytokine-driven tissue injury [192,193] [Evidence: strong]. In parallel, ROS- and replication stress-induced DNA lesions activate the DNA damage response (DDR) network, including ATM/ATR signaling, p53-orthologous activation, and checkpoint kinase engagement, which detect lesions and either coordinate repair or (when damage is overwhelming or repair transcriptionally suppressed) trigger apoptosis [129] [Evidence: moderate]. The replication stress signature discussed in Section 6.3 is a particularly informative ATR–Chk1-driven component of DDR engagement and currently rates as emerging evidence in invertebrate NP/MP work, given that direct mechanistic markers (phospho-Chk1, RPA32 phosphorylation, ssDNA accumulation) are seldom assayed. Inflammation and DDR are not independent: inflammatory ROS contribute to the lesion burden that DDR must address, while persistent DDR signaling activates inflammatory transcriptional programs through NF-κB. The two cascades therefore reinforce one another and feed jointly into the epigenetic integration described next.

### 12.6. Epigenetic Propagation and Adverse Outcome Integration

Oxidative stress, DDR signaling, and inflammatory transcriptional programs converge on the epigenome through several documented mechanisms: ROS-driven oxidation of methyl-CpG and of methylation-machinery cofactors; DDR-coupled recruitment of chromatin modifiers to damaged loci; and inflammatory-cytokine-driven shifts in histone-modification landscapes. NP/MP exposure produces altered DNA methylation, histone modifications, and miRNA expression in invertebrate models consistent with these inputs [181,194], and at least one study has provided RNAi-based causal validation that methylation-related factors (*met-2*, *set-2*, *spr-5*) are required for transgenerational transmission of NP-induced phenotypes [182] [Evidence: moderate, but restricted to *Caenorhabditis elegans*]. We emphasize that this moderate rating reflects causally interrogated evidence in a single, exceptionally tractable nematode model; for macroinvertebrates (bivalves, *Daphnia*, earthworms), stable transgenerational epigenetic inheritance following NP/MP exposure is at best emerging and largely uncorroborated, and the term “transgenerational” should not be applied to the predominant F0–F1(–F2) multigenerational designs in those taxa. The functional consequence is that epigenetic remodeling can reshape the transcriptional landscape and may sustain stress-response or maladaptive states beyond the exposure window, and (in a small number of experimental systems) into one or two offspring generations. Whether such marks support stable transmission into generations beyond F2 in invertebrate NP/MP exposures remains to be validated. Across the levels described in Section 12.3, Section 12.4, Section 12.5 and Section 12.6, the architecture is best understood not as a strict linear cascade but as a network in which oxidative, mitochondrial–lysosomal, ER stress, inflammatory, DDR, and epigenetic events reinforce one another; this network framing accommodates the heterogeneity of effect directions reported across studies (Section 14) without invoking inconsistency where none exists. The network terminates in individual-level adverse outcomes (reduced fecundity, developmental abnormalities, behavioral impairment) and, with diminishing evidentiary support, in multigenerational fitness consequences and inferred but currently unvalidated population- and community-level effects. The evidence-strength stratification of these adverse outcome tiers is examined in Section 12.7.

### 12.7. Summary of Evidence Strength Along the AOP

Across the AOP architecture proposed here, the strength of supporting evidence varies systematically with proximity to the molecular initiating event. At the MIE and proximal key-event level, evidence is strong for direct membrane interaction (MIE-1), corona-mediated uptake (MIE-2), ROS as a recurrent downstream node, mitochondrial dysfunction, lysosomal destabilization, and inflammatory signaling; it is moderate for surface-catalyzed redox chemistry (MIE-3), Trojan horse delivery (MIE-4), ER stress, and DDR engagement. At the molecular damage level, evidence is strong for oxidative DNA lesions, micronucleus formation, and apoptosis induction; it is emerging for replication stress as a discrete mechanism, for telomere damage, for DNA–protein crosslinks, and for the mitochondrial–lysosomal feedback loop in invertebrate models. At the adverse outcome level, evidence is strong for impaired fecundity, developmental abnormalities, and behavioral deficits at the individual scale; moderate for multigenerational fitness loss within the directly exposed (F0) and germline-exposed (F1, occasionally F2) generations; and emerging and currently indirect for stable transgenerational transmission beyond F2 and for population- and community-level consequences. The further inference that NP/MP exposure drives community structure shifts or biodiversity decline currently rests on mechanistic plausibility rather than on quantitative ecological evidence and should be treated as a research priority rather than as a documented adverse outcome. This stratification serves two purposes. It clarifies where the next generation of mechanistic experiments should be directed, in particular, towards the emerging evidence elements where causal demonstration is currently weakest. It also signals to risk-assessment practitioners which AOP components currently support quantitative inference and which require additional empirical anchoring [195].

## 13. Mechanistic Cross-Study Synthesis: Comparative Patterns of NP/MP Effects Across Invertebrate Models

Table 1 provides a structured cross-study synthesis of representative NP/MP studies in invertebrate models. It aligns species, particle composition, size class, exposure concentration, exposure duration, and observed multi-level effects, and (rather than computing pooled effect sizes) uses qualitative annotations of dominant pathway, exposure realism, and evidence strength to support transparent cross-study comparison. Selection prioritizes mechanistically informative studies that span genotoxic, oxidative, molecular, omics, reproductive, and transgenerational endpoints. The synthesis is illustrative rather than exhaustive. Its aim is to enable explicit comparison of NP- versus MP-associated effect profiles, of polymer-specific patterns, and of environmentally realistic versus experimentally inflated exposure regimes, in a form that complements the AOP architecture of Section 12 without claiming the inferential weight of a formal meta-analysis.

### Environmentally Relevant Versus Experimental Concentrations: A Critical Appraisal

A persistent and pivotal critique of NP/MP ecotoxicology is the discordance between exposure concentrations used in laboratory studies and those measured in environmental compartments. Reported environmental MP concentrations span approximately 10^−3^–10^2^ particles L^−1^ in surface waters, with sediment values reaching 10^2^–10^3^ particles kg^−1^ in heavily impacted areas. NP concentrations are even less well constrained owing to detection limitations. Indirect estimates suggest sub-µg L^−1^ to low-µg L^−1^ levels in many surface waters, with localized hotspots potentially higher [4,33,63,64].

By contrast, the majority of laboratory studies summarized in Table 1 employ exposures in the mg L^−1^ to g L^−1^ range. Such doses exceed median environmental concentrations by three to six orders of magnitude. Inflated exposures can be defended as worst-case-scenario tools for hazard identification. They nevertheless compromise extrapolation to real-world risk and often conflate dose-related effects with experimental artifacts, particle aggregation, food dilution, and surfactant carryover [21,63]. A growing number of studies (e.g., [42,43,60,61,180]) employ environmentally realistic exposures in the µg L^−1^ range. These still report measurable molecular and reproductive effects, particularly under chronic and multigenerational designs. The findings undermine the assumption that environmentally realistic concentrations are necessarily innocuous and reinforce the importance of duration- and life-stage-resolved exposure designs.

A further interpretive caution concerns the nature of the responses recorded at the very highest exposures. We adopt ≥1 g L^−1^ as an operational upper bound above which observed cytotoxicity should not, on its own, be interpreted as evidence for a polymer-specific molecular pathway. At mg/L to g/L loadings, sub-micron beads reach per-cell particle numbers that are orders of magnitude above anything encountered in the field; for example, at 10–15 mg/L of 50-nm beads a single hemocyte or *Daphnia* cell is confronted with an enormous particle burden. Under these conditions, the two endpoints most frequently invoked as evidence of polymer-specific cytotoxicity—collapse of mitochondrial membrane potential and frank plasma-membrane lysis (LDH leakage, propidium-iodide uptake)—are most parsimoniously attributed to non-specific physical overload, particle crowding, surface occlusion, and effective cellular suffocation, rather than a polymer-specific molecular pathway, and would be reproduced by inert particles of comparable size and number. Accordingly, any effect reported only at ≥1 g L^−1^ is classified in this review as a non-specific physical-occlusion signal and is excluded from the polymer-specific mechanistic interpretation of the AOP in Section 12, irrespective of the biochemical readout used. Effects observed only at such concentrations should therefore be read as upper-bound hazard signals and must not be mapped onto the AOP as environmentally operative mechanisms without independent support at realistic loadings. Throughout this review, AOP links anchored only in high-concentration data are flagged accordingly (see the evidence-strength stratification in Section 12.7 and the exposure concentration annotation in Table 1). The same structural concern has been raised independently for the herpetofaunal literature, in which experimental bias toward acute, supra-environmental laboratory exposures using pristine, single-polymer particles has been identified as a major obstacle to translating laboratory findings into population-level risk characterization for amphibians and reptiles [25]. The convergence of this critique across vertebrate and invertebrate syntheses indicates that the concentration tier and weathering state caveats applied here are a generic feature of the current MP toxicology literature rather than a peculiarity of invertebrate work.

The interpretive implication is twofold. Mechanistic understanding gained at high concentrations remains valuable for hazard identification and pathway elucidation. Risk-relevant inference, however, requires matched exposure realism. The field would benefit from explicit reporting of particle number concentrations alongside mass concentrations, from systematic chronic studies at sub-mg L^−1^ exposures, and from concentration–response anchoring against measured environmental distributions for invertebrate habitats.

## 14. Critical Analysis: Conflicting Findings, Methodological Pitfalls, and Model Limitations

The NP/MP invertebrate ecotoxicology literature is rich but fragmented. A critical layer of analysis is therefore essential to convert empirical heterogeneity into mechanistic insight. Three categories of inconsistency dominate: divergent effect directions for the same endpoint; non-replication of effects across laboratories using nominally similar particles; and apparent contradictions between high-concentration acute and low-concentration chronic exposure regimes; as detailed in Section 13, effects reported only at supra-environmental loadings (≥1 g L^−1^) are classified in this review as non-specific physical-occlusion signals rather than polymer-specific pathway activation. Each is mechanistically interpretable when particle physicochemistry, dispersion stability, and tissue context are properly resolved.

### 14.1. Conflicting Findings and Their Mechanistic Interpretation

#### 14.1.1. Biphasic Antioxidant Enzyme Responses

Reports of SOD, CAT, GPx, and GST induction in some studies and inhibition in others have been treated as contradictory. Closer examination reveals a consistent biphasic pattern: induction at low-to-moderate doses and short durations, transitioning to inhibition at high doses that approach the non-specific physical-occlusion regime defined in Section 13, as adaptive transcriptional capacity is overwhelmed, and oxidative inactivation of the enzymes themselves becomes dominant [145,146]. The biphasic pattern is mechanistically informative, not contradictory. It supports the use of time-resolved sampling to distinguish adaptive from maladaptive regimes.

#### 14.1.2. Opposing DNA Methylation Directions

Reports of both global hypermethylation and hypomethylation following NP exposure in invertebrates initially appear inconsistent [43,161,162]. The conflict resolves when polymer chemistry, exposure duration, and tissue context are considered. Acute high-dose exposure tends to produce locus-specific hypermethylation of stress-responsive promoters consistent with transcriptional silencing of housekeeping genes. Chronic low-dose exposure produces global hypomethylation consistent with one-carbon metabolism depletion (depleted SAM pools, GSH-driven sulfur diversion). The two patterns are not contradictory but reflect distinct mechanistic regimes operating at different exposure intensities.

#### 14.1.3. Contradictory Micronucleus and Comet Assay Outcomes

Some studies report robust micronucleus induction at concentrations where others find no effect, even using ostensibly similar particles. The dominant explanation lies in particle dispersion stability: aggregated particles behave as effective microparticles regardless of nominal nano-scale labeling [23,63]. The role of fluorescent labels and surfactant preservatives in confounding apparent biological effects has been clearly demonstrated [21,23]. Any study that does not report hydrodynamic diameter, polydispersity, and dispersion-medium stability should therefore be interpreted with caution.

### 14.2. Methodological Pitfalls and Sources of Inter-Study Variability

Beyond mechanistic interpretation, several methodological issues account for substantial inter-study variability. First, particle characterization is frequently incomplete. Studies often report nominal manufacturer specifications rather than as-tested hydrodynamic size, zeta potential, polydispersity, and corona composition in the actual exposure medium [26,27,63]. Second, dispersion protocols (sonication regime, dispersant choice, ionic strength of medium) vary widely. They substantially affect particle aggregation behavior and often determine whether organisms are exposed to true nano-scale or to effectively microscale aggregated material. Third, exposure media differ in ionic strength, dissolved organic matter, and natural organic corona constituents. All of these modulate particle behavior and bioavailability. Fourth, fluorescently labeled particles can release dye into solution, generating apparent uptake signals that do not reflect particle internalization [23]. Fifth, commercial particle preparations may contain residual surfactants and preservatives whose biological effects are non-trivial [21]. This confound is especially important for cationic, amine-functionalized polystyrene (PS-NH_2_): some of the membrane destabilization, lactate dehydrogenase leakage, and acute hemocyte mortality attributed to these particles may be driven, wholly or in part, by the surfactants and dispersants used during synthesis to prevent irreversible aggregation in the stock suspension, rather than by the polymer surface itself. Relatively few primary studies document dialysis of their particles or include surfactant-only (particle-free vehicle) controls, and the present review was not able to verify the presence of such controls systematically across all included studies. Cytotoxicity attributed specifically to cationic nanoplastics should therefore be interpreted with corresponding caution, and future work should report dialysis and surfactant-only controls as standard. The cumulative effect of these methodological inconsistencies is that nominal mass concentrations across studies are not directly comparable. Apparent inter-study contradictions often reflect uncontrolled physicochemical heterogeneity rather than genuine biological discordance [38].

### 14.3. Limitations of Current Invertebrate Models

Each commonly used invertebrate model carries specific limitations that constrain interpretation. *Daphnia magna* offers superb tractability for life-history endpoints. It is, however, prone to particle entrapment in the carapace and brood pouch, which can produce apparent body burdens unrelated to actual cellular uptake [23]. *Caenorhabditis elegans* provides unparalleled genetic resolution and transgenerational tractability. Its bacterial diet and laboratory medium nevertheless poorly resemble natural soil exposure, limiting environmental extrapolation. Marine bivalves offer ecologically relevant filter-feeding exposure. Their slow generation times complicate transgenerational designs, and their genetic resources (although improving) lag behind those of model nematodes and arthropods. *Eisenia* spp. are central to soil ecotoxicology. Exposure regimes vary widely between paper-contact tests and soil-incorporation tests, with very different bioavailability profiles. *Chironomus* spp. provide sediment-relevant exposure and uniquely tractable polytene chromosomes. Assays, however, are seldom standardized across laboratories. *Artemia* spp. tolerate a broad salinity range and are easy to maintain. Their phylogenetic distance from most other ecotoxicological models complicates cross-species inference. No single model can capture the full taxonomic diversity of invertebrate responses. A multi-species comparative approach (anchored in conserved molecular pathways but explicit about ecological role) is essential, particularly for translating laboratory findings to community- and biodiversity-level risk.

### 14.4. Implications for Interpreting the Literature

The overarching implication is that the NP/MP invertebrate literature should be read with explicit attention to particle physicochemistry, dispersion behavior, exposure realism, and model-specific constraints. Apparent contradictions between studies are often more informative than they first appear. They illuminate boundary conditions and mechanistic regimes that would be invisible in any single study. The need is not for further proliferation of single-endpoint, single-particle studies but for systematic, mechanistically anchored designs that explicitly resolve the determinants of observed variability. Section 16 (Future Perspectives) returns to this point with concrete recommendations.

## 15. Emerging Biomarkers

Ecological risk assessment requires robust biomarkers. While classical biomarkers remain valuable, NP/MP toxicity demands biomarkers capturing molecular and long-term effects [196].

Omics-derived biomarkers offer particular promise. Transcriptomic signatures of oxidative, immune, and DNA damage response pathways; proteomic profiles of carbonylated and ubiquitinated proteins; and metabolomic fingerprints of perturbed energy and lipid metabolism collectively provide multi-dimensional readouts of exposure and effect [43,47,161,178,197]. Epigenetic biomarkers (locus-specific DNA methylation and miRNA signatures) are especially valuable because of their persistence and their potential for cross-generational inference [33,165,182].

Mitochondrial membrane potential, lysosomal membrane stability, γ-H2AX foci, and 8-oxodG levels are mechanistically informative biomarkers that bridge molecular and cellular scales. Behavioral biomarkers, altered swimming patterns in *Daphnia*, locomotor and chemotaxis defects in *C. elegans*, and burrowing behavior in earthworms, integrate neurotoxic, sensory, and physiological perturbations and have been proposed as endpoints with potential relevance for ecological risk assessment [198,199]; their direct linkage to community-level outcomes nevertheless remains to be validated. The development of cross-species biomarker panels, anchored in conserved molecular pathways (e.g., the Nrf2–Keap1 antioxidant axis, conserved DDR components, and core apoptotic regulators), would substantially advance comparative invertebrate ecotoxicology and could, in time, support monitoring designs spanning multiple taxa and environmental contexts.

### Which Invertebrate Taxa Are Most Suitable as NP/MP Bioindicators?

No single invertebrate serves as a universal sentinel; suitability depends on the environmental compartment and monitoring goal. In marine and coastal waters, sessile bivalves (*Mytilus*, Crassostrea) are the most effective biomonitors due to their filtration capacity, wide distribution, and well-established hemocyte-based genotoxic and immunotoxic endpoints, with relevance to seafood exposure [51,60,97]. In freshwater systems, *Daphnia* is widely used for life-history, reproductive, and multigenerational endpoints, although particle entrapment may bias body-burden estimates [23,200]. For sediments, *Chironomus* enables cytogenetic analyses via polytene chromosomes, supporting sensitive genotoxicity assessment [55]. In soils, earthworms (*Eisenia*) remain the standard sentinel integrating dermal and dietary exposure [18,54]. *Caenorhabditis elegans* is more appropriate for mechanistic and transgenerational studies than field biomonitoring. Overall, a compartment-specific, multi-species approach anchored in conserved molecular endpoints (e.g., Nrf2/SKN-1 axis, DNA damage responses, micronucleus frequency) provides the most robust strategy.

## 16. Research Gaps

Despite rapid expansion of the field, several substantial gaps constrain the mechanistic and translational maturity of NP/MP invertebrate toxicology. The gaps below build on the critical analysis of Section 14 and are organized in order of strategic priority for the next phase of research.

### 16.1. Particle Realism

The overwhelming reliance on pristine, monodisperse, fluorescently labeled polystyrene particles produces results of limited environmental relevance. Field-encountered particles are heterogeneous in polymer composition, shape, size distribution, and weathering state [63]. Studies employing environmentally weathered particles, secondary MPs derived from real-world plastic items, and complex particle mixtures remain comparatively rare. They represent a top-priority methodological frontier.

### 16.2. Exposure Realism

Exposure concentrations frequently exceed environmentally realistic levels by several orders of magnitude, raising concerns about ecological extrapolability [64,200]. Conversely, chronic low-dose effects may be missed because of the reliance on short-term, high-dose acute exposure paradigms. Systematic chronic exposure studies anchored against measured environmental concentration distributions are needed.

### 16.3. Underexplored Genotoxic Endpoints

Sister chromatid exchange, DNA–protein crosslinks, telomere damage, and replication stress markers remain markedly underexplored in invertebrate models. Systematic incorporation of these endpoints would substantially expand mechanistic resolution beyond the dominant comet/micronucleus paradigm.

### 16.4. Multi-Omics Integration

Transcriptomic studies are increasingly common, but integrated multi-omics analyses are still limited. Causal frameworks linking molecular initiating events to organismal outcomes are seldom explicitly tested [201]. The gap is conceptual as well as technical: studies often report omics datasets without committing to mechanistic causal claims.

### 16.5. Mixture and Trophic-Transfer Toxicology

The role of particle additive–contaminant mixtures, biofilm-modified particles, and trophic transfer in shaping toxicity is incompletely understood. The “Trojan horse” hypothesis is widely invoked but requires more rigorous quantitative validation across realistic exposure scenarios [186]. Trophic transfer of NPs and MPs through invertebrate-dominated food webs is a mechanistically plausible route by which particle exposure could propagate to higher trophic levels; the quantitative consequences for community composition and biodiversity remain to be validated.

### 16.6. Methodological Standardization

Heterogeneity in particle characterization, dispersion protocols, exposure media, and endpoint quantification undermines cross-study comparability and meta-analytical synthesis [202]. International quality criteria are emerging [27] but remain inconsistently adopted.

### 16.7. True Transgenerational Designs

Transgenerational effects are increasingly discussed but rarely investigated beyond two generations. The mechanistic underpinnings of inherited phenotypes (particularly the relative contributions of germline epigenetic marks, parental provisioning, and microbiome transmission) remain inadequately resolved. As a consequence, projections of long-term population trajectories under chronic NP/MP contamination should at present be treated as theoretical scenarios rather than as validated risk forecasts transgenerational epigenetics has, in parallel, been highlighted as one of the most tractable research priorities in MP ecotoxicology for amphibians and reptiles [25], indicating that the same mechanistic question—whether NP/MP-induced epigenetic marks transmit stably across unexposed generations and translate into demographic-scale consequences—is currently under-resolved on both sides of the vertebrate/invertebrate divide.

### 16.8. Population, Community, and Biodiversity Scales

Most current evidence resides at the individual-organism level. Robust extrapolation from molecular and cellular endpoints to population dynamics, community composition, and ecosystem services is currently more aspirational than achievable; closing this scale gap, through coupled laboratory and field designs and fit-for-purpose population models, is essential before the field can claim genuinely biodiversity-relevant risk assessment.

## 17. Future Perspectives

Advancing the field will require concerted action across the gaps identified in Section 16. Standardization of particle characterization, exposure protocols, and endpoint quantification is foundational, and emerging international guidelines should be widely adopted [203,204]. Beyond standardization, three converging axes are central: greater use of environmentally realistic particles (weathered, heterogeneous, additive- and contaminant-laden) within AOP-anchored designs; integrated multi-omics combining transcriptomic, proteomic, metabolomic, and epigenomic readouts under unified experimental designs and analyzed with network and pathway-based tools to support causal MIE–key-event–adverse outcome chains; and extension of transgenerational designs beyond F2 in clonal *Daphnia* and *C. elegans* to test rigorously for inherited phenotypes and their epigenetic underpinnings.

Integration of in vivo, in vitro, and in silico approaches under a systems toxicology paradigm may, over time, enable predictive modeling of NP/MP risk across species and exposure scenarios; the realism of such models will depend on their being explicitly calibrated against empirical population-level data, which are at present scarce. High-throughput in vitro assays using primary invertebrate cell cultures, organoids, and gene-edited cell lines can complement organismal studies, while computational toxicology, machine-learning-based predictive frameworks, and quantitative AOPs offer increasingly available tools for extrapolation and risk prediction [205,206,207]; their outputs at the population scale should be presented with explicit uncertainty rather than as risk forecasts. Whether scaling can ultimately support biodiversity-relevant inference is itself a research question rather than an assumption. Translation into regulatory action will require continued dialogue between toxicologists, ecologists, polymer chemists, and policymakers, anchored by mechanistic rigor, ecological realism, and calibrated uncertainty about extrapolations beyond the scales at which data exist.

## 18. Conclusions

The available evidence indicates that NPs and MPs exert multi-level toxicological effects on invertebrate animals, but mature interpretation of that evidence requires a shift from descriptive endpoint cataloging toward mechanistically integrated, AOP-anchored analysis. We close with three mechanistic insights, three persistent gaps, and three priorities that, in our reading, will shape the next decade of NP/MP invertebrate ecotoxicology.

### 18.1. Three Mechanistic Insights

First, NP/MP toxicity is best conceptualized as the product of several primary molecular initiating events—direct membrane interaction, protein corona formation, surface-catalyzed redox chemistry, and Trojan horse co-contaminant delivery—whose relative contributions depend on particle physicochemistry, weathering state, and biological context. Trojan horse delivery is best understood as a toxicokinetic transport step that potentiates a downstream chemical initiating event (binding of the delivered metal or organic contaminant to its target), rather than as an initiating molecular interaction in its own right. Treating these MIEs as parallel but resolvable, rather than collapsing them into a single phenomenon, enables both mechanistic interpretation and tiered regulation.

Second, ROS generation, mitochondrial dysfunction, lysosomal destabilization, and ER stress appear to function as a coupled organelle–stress axis through which the MIEs converge on common downstream key events: genotoxicity, apoptosis, autophagy, inflammation, and epigenetic remodeling. Oxidative stress is most defensibly framed not as a sole primary driver, nor as a mere downstream consequence, but as a frequently engaged, non-obligatory node within a multi-input mechanistic network; mechanical injury, membrane wrapping, lysosomal and ER stress, and additive- or co-contaminant-mediated effects can reach downstream key events without passing through a ROS step.

Third, epigenetic remodeling (DNA methylation changes, altered histone marks, and miRNA dysregulation) provides a mechanistically plausible substrate by which acute molecular damage may be propagated into multigenerational phenotypes, and in some experimental systems into the first one or two offspring generations. Whether such marks support stable inheritance across multiple unexposed generations in invertebrate NP/MP exposures, and whether the resulting phenotypic carryover has demographic consequences in natural populations, remain open empirical questions. The proposition warrants more systematic incorporation into NP/MP research designs and risk-assessment thinking than current practice affords, while remaining open to revision as transgenerational evidence matures.

### 18.2. Three Persistent Research Gaps

Three persistent research gaps remain. First, studies largely rely on pristine, monodisperse, fluorescent polystyrene at concentrations far above environmental levels, limiting ecological relevance despite mechanistic insight. Second, key endpoints such as replication stress, DNA–protein crosslinks, sister chromatid exchange, telomere dynamics, and multi-omics integration are still underrepresented, leaving important gaps in AOP frameworks. Third, true transgenerational studies beyond F2 remain scarce, and the relative roles of germline epigenetic inheritance, parental provisioning, and microbiome transmission in multigenerational effects remain unresolved.

### 18.3. Three Future Research Priorities

First, systematic adoption of standardized particle characterization, environmentally realistic exposure regimes (including weathered particles, mixture exposures, and sub-mg L^−1^ chronic doses), and harmonized endpoint protocols, with explicit reporting of particle number alongside mass concentrations.

Second, integrated multi-omics, AOP-anchored experimental designs that explicitly test causal chains from primary MIEs to adverse outcomes, supported by cross-species biomarker panels grounded in conserved molecular pathways.

Third, expansion of multigenerational and systems toxicology studies combining in vivo, in vitro, and in silico approaches, with the medium-term goal of supporting hypothesis-driven, uncertainty-quantified inference from molecular initiating events toward invertebrate population dynamics. Whether such inference can be extended robustly to community-level outcomes will depend on parallel ecological monitoring and on validation against field data, and should at present be treated as a research aspiration rather than a near-term deliverable.

Plastic contamination is unlikely to abate in the near term. Invertebrates are central to animal biodiversity and underpin processes (decomposition, primary consumption, prey provision) that, in well-studied ecosystems, support higher trophic levels. Mechanistically rigorous, AOP-anchored invertebrate ecotoxicology, combined with the methodological honesty that explicitly distinguishes experimentally demonstrated findings, inferred mechanisms, and theoretical ecological implications, will accordingly remain a productive frontier.

## Figures and Tables

**Table 1:** Representative studies of nano- and microplastic effects in invertebrate model organisms. Entries are ordered by taxonomic group and presented as a structured comparative synthesis. Each study is annotated for the dominant mechanistic pathway, for the exposure concentration tier relative to measured environmental concentrations (environmentally relevant ≤ ~10 µg L^−1^ aqueous or comparable sediment loading; moderate = ~10 µg^−1^ mg L^−1^; high = ≥1 mg L^−1^; very high = ≥1 g L^−1^). Note that “high” and “very high” denote concentrations that exceed environmental levels (low ecological realism, appropriate to worst-case hazard identification) rather than a high degree of environmental realism; this concentration tier annotation is therefore fully consistent with the section Environmentally Relevant Versus Experimental Concentrations: A Critical Appraisal, and for qualitative evidence strength (strong, moderate, emerging) applied at the per-study level using the four-criterion convention defined in Section 12. The per-study annotation reflects evidence strength within the cited study system and does not generalize across taxa; aggregated tier assignments across pathway components appear in Section 12.7. A dedicated column reporting whether each study documented a dialysis step or a surfactant-only vehicle control has been added to flag a potential source of confounding in the comparative table; because the present review did not systematically extract this information from the primary literature, all entries are annotated as “Not reported”, with a supplementary ‡ marker for cationic PS-NH_2_ preparations for which this omission is analytically most consequential (see Section 14.2).

Species	Particle (Polymer/State)	Size	Conc. & Duration	Dominant Pathway	Key Outcome	Concentration Tier (vs. Environmental)	Evidence	Dialysis/Surfactant-Only Vehicle Control Reported?	Refs.
*Daphnia magna*	PS-NH_2_ (cationic NP)	52 nm	0.1–10 mg L^−1^; 21 d	Endocrine/reproductive	↓ fecundity, ↓ neonate size, vitellogenin/ecdysone-receptor dysregulation	High	Strong	Not reported ‡	[39,40]
*Daphnia magna*	PS-COOH (anionic NP)	70 nm	0.32–32 µg L^−1^; 21 d (F0–F3)	Epigenetic/multigenerational (F0–F3)	Multigenerational fitness depression; altered methylation of stress loci	Env-relevant	Moderate	Not reported	[41,42]
*Daphnia pulex*	PS-NP	75 nm	0.1–1 mg L^−1^; 21 d	Metabolic/immune	Glycometabolism and immune-defense dysregulation; ↑ SOD, ↑ MDA	High	Moderate	Not reported	[43]
*Caenorhabditis elegans*	PS-NP	30, 100 nm	1 µg L^−1^–10 mg L^−1^; acute–chronic, F1–F10	Neuro-genotoxic/transgenerational	Size-dependent neurotoxicity, intestinal damage, oxidative DNA damage, transgenerational fitness loss	Variable	Strong	Not reported	[44,45,46]
*Caenorhabditis elegans*	PS-NP	100 nm	10 µg L^−1^; L1–L4	ER stress/UPR	UPR activation, p38 MAPK signaling, germline apoptosis (*ced-3*/*ced-4*)	Env-relevant to moderate	Strong	Not reported	[47,48]
*Mytilus galloprovincialis*	PS-NH_2_ (cationic NP)	50 nm	1–50 mg L^−1^; 24–96 h	Apoptotic/mitochondrial	Hemocyte apoptosis, lysosomal destabilization, mitochondrial potential collapse	High	Strong (acute)	Not reported ‡	[49,50]
*Mytilus galloprovincialis*	PE-MP (irregular)	20–25 µm	1.5 g L^−1^; 7 d	Digestive/lysosomal	Histological alterations, lysosomal stress, oxidative imbalance	Very high	Moderate	Not reported	[51]
*Mytilus* spp.	PS-MP + PAHs	20 µm	1.5 µg L^−1^ + 32 µg L^−1^ PAHs; 7 d	Trojan horse/genotoxic	PAH bioaccumulation; ↑ comet tail moment, ↑ MN frequency	Moderate (PAH-relevant)	Moderate	Not reported	[52,53]
*Eisenia fetida*/*andrei*	PS-MP	250 µm	62.5–1000 mg kg^−1^ soil; 28 d	Genotoxic/immune	Coelomocyte DNA damage, histopathology, immune gene dysregulation	High	Strong	Not reported	[18]
*Eisenia fetida*	LDPE-MP (aged)	<150 µm	0.1–1.5% *w*/*w*; 14–28 d	Oxidative/microbiome	↓ growth, ↑ SOD/CAT/MDA, altered gut microbiome	High	Moderate	Not reported	[54]
*Chironomus riparius*	PE-MP (irregular)	32–63 µm	0.5 g kg^−1^ sediment; larval stage	Cytogenetic	Mouthpart deformities, polytene chromosome aberrations, ↓ emergence	High (realistic for impacted sediments)	Strong	Not reported	[55,56]
*Artemia franciscana*	PS-NP (anionic)	40 nm	0.001–10 mg L^−1^; 48 h	Behavioral/oxidative	Altered swimming, ↑ ROS, ↓ molting	Variable	Moderate	Not reported	[57]
*Artemia salina*	PS-NP	100 nm	1–10 mg L^−1^; 24–96 h	Apoptotic/oxidative	ROS accumulation, caspase-3 activation, antioxidant enzyme imbalance	High	Moderate	Not reported	[58]
*Brachionus koreanus*	PS-MP	0.05, 0.5, 6 µm	0.1–20 µg mL^−1^; 12–24 h	Oxidative/MAPK	Size-dependent oxidative stress, p-JNK/p-p38, MAPK–Nrf2 modulation	Moderate to high	Strong	Not reported	[59]
*Crassostrea gigas*	PS-MP	2, 6 µm	0.023 mg L^−1^; 2 months	Reproductive	↓ oocyte number, ↓ sperm velocity, offspring growth depression	Env-relevant	Strong	Not reported	[60]
*Hediste diversicolor*	PE-MP (env-realistic)	5–250 µm	0.008 mg L^−1^; 21 d	Immune/oxidative	Immunotoxicity and oxidative stress at environmentally realistic exposure	Env-relevant	Strong	Not reported	[61]
*Ruditapes philippinarum*	PS-MP + Hg	1, 50 µm	0.1–1 mg L^−1^ + 5 µg L^−1^ Hg; 14 d	Mixture/Trojan horse	Synergistic immunomodulation, histopathology, oxidative stress	Moderate (Hg-relevant)	Moderate	Not reported	[62]

Abbreviations: PS, polystyrene; PE, polyethylene; LDPE, low-density polyethylene; PVC, polyvinyl chloride; PAHs, polycyclic aromatic hydrocarbons; NP, nanoplastic; MP, microplastic; SOD, superoxide dismutase; CAT, catalase; MDA, malondialdehyde; MN, micronucleus; UPR, unfolded protein response; PS-NH_2_, amine-functionalized (cationic) polystyrene; PS-COOH, carboxylate-functionalized (anionic) polystyrene. Notes: the “Exposure realism” column is a qualitative judgment made relative to current best estimates of environmental NP/MP concentrations [4,33,63,64] and should be revised as monitoring data improve. † Aqueous exposures ≥1 g L^−1^. Per the section Environmentally Relevant Versus Experimental Concentrations: A Critical Appraisal, effects observed only at such loadings are classified as non-specific physical-occlusion signals; the “Dominant pathway” entry for such rows does not represent a polymer-specific molecular pathway and is retained only for historical comparability with the primary literature. ‡ Cationic (PS-NH_2_) preparations are the class most vulnerable to surfactant-driven artifacts in cytotoxicity readouts (see Section 14.2). None of the cited PS-NH_2_ studies in this table explicitly reports either a dialysis step or a surfactant-only vehicle control; cytotoxic effects attributed to these particles—including hemocyte apoptosis, lysosomal destabilization, and mitochondrial membrane potential collapse—should therefore be interpreted with corresponding caution and should not, on the strength of these studies alone, be regarded as unambiguously polymer-specific.

**Table 2:** Representative gene-expression responses to NP/MP exposure reported across invertebrate models, organized by functional category. The table consolidates gene-level responses described in Section 6, Section 7 and Section 8 and Section 10 and is intended to assist the selection of physiological and molecular markers; it summarizes the direction and taxonomic occurrence reported in the cited primary literature and does not constitute a quantitative meta-analysis. Gene symbols follow the conventions used in the cited studies.

Gene/Gene Family	Function/Pathway	Representative Invertebrate Taxa	Typical Direction	Refs.
*hsp70*, *hsp90*	Proteostasis/heat-shock response	*Daphnia*, *Mytilus*, *C. elegans*	Up (induced)	[129,130,131]
Metallothioneins	Metal binding/detoxification	Mollusks and other taxa	Altered	[129,130,131]
Cytochrome P450 (CYP)	Xenobiotic biotransformation	Multiple taxa	Altered	[129,130,131]
SOD, CAT, GPx	Enzymatic antioxidant defense	*Daphnia*, *Artemia*, *Eisenia*, *mollusks*	Biphasic (induction then inhibition)	[43,51,58,61,119,120,121]
GST/*glutathione*-related	Phase-II detoxification/antioxidant	Multiple taxa	Altered/biphasic	[51,61,126]
Nrf2–Keap1 (SKN-1 in *C. elegans*)	Antioxidant/detoxification transcription	*C. elegans*, *Mytilus*	Up (activated)	[148,149]
*vitellogenin*	Reproduction/yolk provisioning	*Daphnia*	Dysregulated	[40,150]
*ecdysone*-recepto*r*/*ecdysone*-related	Molting and reproductive signaling	*Daphnia*	Dysregulated	[40,150]
*chitin-metabolism* genes	Cuticle formation/development	*Daphnia*	Altered	[150]
*ced-3*, *ced-4*, *egl-1*	Germline apoptosis	*C. elegans*	Up	[47,48]
Bax/Bcl-2	Apoptotic balance	Marine bivalves	Pro-apoptotic imbalance	[52]
*ogg1*, *ape1*	Base excision repair (BER)	*Daphnia*, *C. elegans*, *Mytilus*	Altered	[52,114,115,130,137]
*xpa*, *xpc*	Nucleotide excision repair (NER)	*Daphnia*, *C. elegans*, *Mytilus*	Altered	[52,114,115,133,137]
*msh2*, *mlh1*	Mismatch repair (MMR)	*Daphnia*, *C. elegans*, *Mytilus*	Altered	[52,114,115,128]
*rad51*, *brca1-like*	Double-strand break/HR repair	*Daphnia*, *C. elegans*, *Mytilus*	Altered (often up)	[52,114,115,128]
*bip*, *chop*, *xbp1*	ER stress/unfolded protein response	*C. elegans*, *Mytilus*	Up	[48]
*lysozyme*, *AMPs*, *TLR* pathway, *NF-κB* orthologs	Innate immunity/inflammation	Mollusk hemocytes, earthworm coelomocytes	Dysregulated	[32,50]
conserved *miRNAs*	Post-transcriptional regulation	*Mollusks*, *Daphnia*	Differential	[151,152,153]

Abbreviations: HR, homologous recombination; AMPs, antimicrobial peptides; TLR, Toll-like receptor; SOD, superoxide dismutase; CAT, catalase; GPx, glutathione peroxidase; GST, glutathione S-transferase. “Altered” denotes consistent dysregulation whose direction varied with species, particle, and exposure regime across the cited studies.

## Data Availability

No new data were created or analyzed in this study. Data sharing is not applicable to this article.

## Abbreviations

The following abbreviations are used in this manuscript:

AOP	Adverse outcome pathway
DDR	DNA damage response
MAPK	Mitogen-activated protein kinase
MDA	Malondialdehyde
MIE	Molecular initiating event
MP	Microplastic
NP	Nanoplastic
ROS	Reactive oxygen species
SOD	Superoxide dismutase
UPR	Unfolded protein response
